# Separacenes A–D, Novel Polyene Polyols from the Marine Actinomycete, *Streptomyces* sp

**DOI:** 10.3390/md11082882

**Published:** 2013-08-13

**Authors:** Munhyung Bae, Heegyu Kim, Yoonho Shin, Byung Yong Kim, Sang Kook Lee, Ki-Bong Oh, Jongheon Shin, Dong-Chan Oh

**Affiliations:** 1 Natural Products Research Institute, College of Pharmacy, Seoul National University, Seoul 151-742, Korea; E-Mails: baemoon89@snu.ac.kr (M.B.); dicafree5@snu.ac.kr (Y.S.); sklee61@snu.ac.kr (S.K.L.); shinj@snu.ac.kr (J.S.); 2 Department of Agricultural Biotechnology, College of Agriculture and Life Science, Seoul National University, Seoul 151-921, Korea; E-Mails: hqhqeori@naver.com (H.K.); ohkibong@snu.ac.kr (K.-B.O.); 3 Division of Agricultural Microbiology, National Academy of Agricultural Science, Rural Development Administration, Suwon 441-707, Korea; E-Mail: greg6044@gmail.com

**Keywords:** marine actinomycete, polyene polyol, cytotoxicity, isocitrate lyase

## Abstract

Separacenes A–D (**1**–**4**), novel polyene polyols, were isolated from *Streptomyces* sp. collected from the southern area of Jeju Island, Korea. The chemical structures of **1**–**4** were established by NMR, mass, UV, and IR spectroscopy as well as the modified Mosher’s method. Separacenes A–B (**1**–**2**), which share an identical planar structure but possess different relative configurations, bear tetraene units flanked by two diol moieties, whereas the stereoisomeric separacenes C–D (**3**–**4**) possess a triene moiety between two diol substructures. Separacenes A–D each contain a terminal olefinic methylene. Separacene A displayed inhibitory activity against *Candida albicans* isocitrate lyase and weak cytotoxicity against both the colon carcinoma cell line HCT-116 and the lung cancer cell line A549.

## 1. Introduction

Bioactive natural products represent a broad frontier in drug discovery [1]. Despite extensive study of the synthesis of bioactive compounds via directed organic and combinatorial methods, only 0.001% of synthetic compounds become drug candidates [2]. A significantly higher proportion (~0.3%) of isolated natural polyketides have been developed as drug candidates [2]. These statistics indicate that natural products have considerable advantages in the discovery and development of new drugs. Thus, natural products and chemical structures derived from or related to natural products play a significant role in pharmaceutical development [3]. 

The secondary metabolites of actinomycetes are prolific sources of bioactive natural products for drug discovery [4]. Numerous actinomycete secondary metabolites have been developed as clinically used drugs, including doxorubicin, amphotericin, rapamycin, vancomycin, and daptomycin. While studies of terrestrial microorganisms tend to yield previously known secondary metabolites, in the last 10 years, marine actinomycetes have been identified as new sources of novel bioactive compounds [5,6]. As a result of several pioneering studies of marine actinomycetes, new drug candidates such as salinosporamide A and thiocoraline are in clinical trials for drug development. This clearly indicates the biomedical potential of marine actinomycetes [7,8]. We recently discovered a new macrocyclic lactone that functions as an isocitrate lyase inhibitor, bahamaolide A, from a *Streptomyces* sp. isolated from the Bahamas [9], and a new lasso peptide, sungsanpin, from another *Streptomyces* sp. collected from deep-sea areas off of Jeju Island, Korea [10].

As part of ongoing efforts to identify new bioactive compounds from marine actinomycetes, we selectively isolated actinomycetes from marine-derived sediment samples collected on seashores and chemically analyzed the cultures for secondary metabolite production. LC/MS analyses indicated that one of the *Streptomyces* strains, SNJ210, isolated in the southern area of Jeju Island, produced polyunsaturated compounds with multiple conjugated double bonds. Here we report the isolation, structural determination (including absolute configurations), and biological activity of four new polyene polyols, separacenes A–D (**1**–**4**).

## 2. Results and Discussion

### 2.1. Structural Elucidation

Separacene A (**1**) was purified as a white powder with the molecular formula C_15_H_22_O_4_, as determined by HR-FAB mass spectrometry (obsd [M + Na]^+^ at *m*/*z* 289.1417, calcd [M + Na]^+^ 289.1416) as well as ^1^H and ^13^C NMR data (Table 1). The ^1^H NMR spectrum of **1** in pyridine-*d*_5_ displayed characteristic polyunsaturated and polyhydroxylated signatures, with 10 olefinic protons between 6.79 and 5.30 ppm and four protons attached to oxygen-bearing carbons between 4.58 and 4.11 ppm. Further analysis of the ^1^H NMR spectrum of **1** identified only one methyl group resonating as a doublet at 1.43 ppm. The ^13^C NMR and gHSQC spectra displayed nine olefinic methine and one methylene carbons from 139.8 to 115.4 ppm, four oxygen-bound methine carbons from 77.3 to 71.2 ppm, and one methyl group at 19.6 ppm. The IR absorption at 3370 cm^−1^ identified some or all of the oxygenated functionalities as hydroxy groups. The identification of 10 olefinic carbons in the ^13^C NMR spectrum and 11 olefinic protons in the ^1^H NMR spectrum indicated that separacene A (**1**) bears five double bonds, accounting for all the unsaturation calculated from the molecular formula. The UV absorption maximum at 302 nm indicated that four of the five double bonds are conjugated.

**Figure 1:**
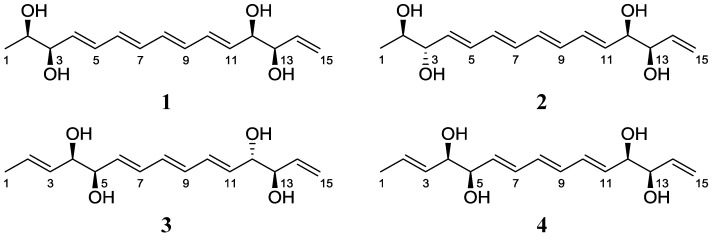
The structures of separacenes A–D (**1**–**4**).

Analysis of the gHSQC spectrum enabled the assignment of all of the one-bond correlations between protons and carbons. The planar structure was then identified and described by ^1^H-^1^H COSY and HMBC NMR spectroscopic analyses. In particular, 2 separate diol moieties were elucidated based on the observed H-2 (δ_H_ 4.11)–H-3 (δ_H_ 4.43) and H-12 (δ_H_ 4.58)–H-13 (δ_H_ 4.54) homonuclear correlations. As predicted by the UV spectrum, four conjugated double bonds (C-4 to C-11) were identified by COSY correlations. The initial acquisition of NMR spectroscopic data at 500 MHz was complicated by intensively overlapped double-bond proton signals and second-order peaks, which hampered the unequivocal establishment of atomic connectivity and double bond geometry. Further NMR analysis at higher field (900 MHz) yielded separated signals and the ^1^H-^1^H coupling constants of the olefinic protons. COSY spectroscopic analysis confirmed the H-11 (δ_H_ 6.26) to H-10 (δ_H_ 6.79) and H-4 (δ_H_ 6.21) to H-5 (δ_H_ 6.75) correlations, permitting the assignment of the C-10–C-11 and C-4–C-5 double bonds. Further COSY analysis revealed the correlation of H-10 with H-9 (δ_H_ 6.44), establishing the C-9–C-10 bond. The connection of C-9 with C-8 was then established by the COSY correlation between H-9 and H-8 (δ_H_ 6.38). The proton H-8 was correlated with H-7 (δ_H_ 6.40), confirming the double bond between C-8 and C-7. Finally, H-7 (δ_H_ 6.40) displayed homonuclear coupling with H-6 (δ_H_ 6.42), completing the assignment of the C-4 to C-11 tetraene structure in separacene A (**1**). The last double bond, C-14–C-15 (an olefinic methylene), was assigned based on the correlation of H_2_-15 (δ_H_ 5.66; 5.30) with H-14 (δ_H_ 6.35) in the COSY spectrum. The first diol (C-2 and C-3) was shown to be flanked by the methyl group (C-1) and the conjugated tetraene (C-4 to C-11) by COSY and HMBC correlations. The connections of the second diol (C-12 and C-13) to both the tetraene and the terminal double bond (C-14–C-15) were also established with these techniques. The gHMBC NMR spectrum supported the assigned structure of **1**. Proton H-9 (δ_H_ 6.44) displayed an HMBC correlation to C-11 (δ_C_ 135.7), and H-11 (δ_H_ 6.26) showed heteronuclear coupling to C-9 (δ_C_ 133.3). In addition, the HMBC correlations from H-3 (δ_H_ 4.43) to C-5 (δ_C_ 131.6) and from H-4 (δ_H_ 6.75) to C-6 (δ_C_ 133.3) supported the C-5–C-6 linkage. Therefore, the gross structure of separacene A (**1**) was elucidated as a linear chain bearing 2 diols, a tetraene unit, and a terminal olefinic methylene. For assignment of the double bond geometries of **1**, the coupling constants in the ^1^H NMR spectrum at 900 MHz were analyzed. The large coupling constants (larger than 15.0 Hz) observed for all the olefinic protons of the tetraene moiety allowed the determination of the configurations as 4*E*, 6*E*, 8*E*, and 10*E* (Figure 1). 

**Figure 2:**
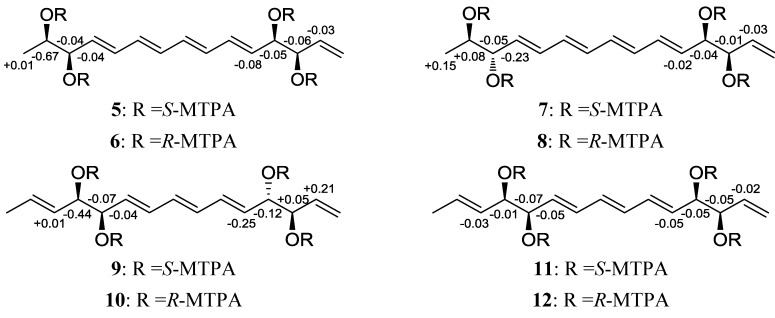
Δδ_S−R_ values of **5**–**12** in pyridine-*d*_5_.

The absolute configurations of the four stereogenic centers of separacene A (**1**) were determined by the modified Mosher’s method for secondary diols [11]. We derivatized separacene A with *R*- and *S*-α-methoxy-α-(trifluoromethyl)phenylacetyl chloride (MTPA-Cl) to yield tetra-*S*- and -*R*-MTPA esters (**5** and **6**), respectively. The ^1^H chemical shifts around the 4 stereogenic centers (C-2, C-3, C-12, and C-13) of **5** and **6** were assigned by ^1^H and COSY NMR analysis. The Δδ*_S_*_−*R*_ values of **5** and **6** displayed sign distributions consistent with *syn*-1,2-diol configurations for the two diol moieties, assigning the absolute configurations as 2*R*, 3*R*, 12*R*, and 13*R* (Figure 2). 

**Table 1:** NMR data for 1 and 2 in pyridine-d5.

C/H	1					2				
	δ_H_^a^	mult (*J* in Hz)		δ_C_^b^			δ_H_^c^	mult (*J* in Hz)	δ_C_^d^	
**1**	1.43	d (6.5)		19.6	CH_3_		1.54	d (5.5)	20.7	CH_3_
**2**	4.11	m		71.2	CH		4.24	dd (11.0, 5.5)	72.6	CH
**3**	4.43	dd (10.0, 6.0)		77.3	CH		4.56	m	77.8	CH
**4**	6.21	dd (15.5, 6.0)		135.9	CH		6.35	dd (15.5, 6.5)	137.8	CH
**5**	6.75	dd (15.5, 10.5)		131.6	CH		6.76	dd (15.5, 9.5)	132.3	CH
**6**	6.42	dd (15.0, 10.5)		133.3	CH		6.44	dd (14.5, 9.5)	133.3	CH
**7**	6.40	m		132.9	CH		6.38	m	133.7	CH
**8**	6.38	m		132.8	CH		6.40	m	134.0	CH
**9**	6.44	dd (15.0, 10.5)		133.3	CH		6.42	dd (15.5, 10.0)	134.3	CH
**10**	6.79	dd (15.5, 10.5)		131.5	CH		6.80	dd (15.5, 10.0)	132.8	CH
**11**	6.26	dd (15.5, 6.0)		135.7	CH		6.26	dd (15.5, 6.0)	136.8	CH
**12**	4.58	dd (9.5, 6.0)		76.0	CH		4.63	dd (11.0, 6.0)	77.3	CH
**13**	4.54	dd (9.5, 5.5)		76.5	CH		4.58	m	78.2	CH
**14**	6.35	ddd (17.5, 10.5, 5.5)		139.8	CH		6.36	ddd (17.5, 10.5, 5.5)	142.4	CH
**15**	5.66	dd (17.5, 1.0)		115.4	CH_2_		5.70	dd (17.5, 1.0)	115.4	CH_2_
	5.30	dd (10.5, 1.0)					5.31	dd (10.5, 1.0)		

^a^ 900 MHz; ^b^ 225 MHz; ^c^ 600 MHz; ^d^ 125 MHz.

Separacene B (**2**) was isolated as a white powder with the molecular formula C_15_H_22_O_4_, as determined by HR-FAB mass spectrometry (obsd [M + Na]^+^ at *m/z* 289.1410, calcd [M + Na]^+^ 289.1416) as well as ^1^H and ^13^C NMR spectroscopy (Table 1). The NMR, IR, mass spectra, and UV data for **2** were almost identical to those for **1**; thus, **2** was expected to be a stereoisomer of **1**. Further analysis of NMR data (^1^H, ^13^C, COSY, HSQC, and HMBC) confirmed the structure of separacene B (Figure 1). The configurations of the double bonds were established as 4*E*, 6*E*, 8*E*, and 10*E* by the ^1^H-^1^H coupling constants. As for compound **1**, the absolute configuration of **2** was determined by MTPA derivatization to yield the tetra-*S*- and -*R*-MTPA esters (**7** and **8**). Calculation of the Δδ*_S_*_−*R*_ values of **7** and **8** established the absolute configurations of the 4 chiral centers as 2*R*, 3*S*, 12*R*, and 13*R* (Figure 2).

Separacene C (**3**) was purified as a white powder with the molecular formula C_15_H_22_O_4_, as determined by HR-FAB mass spectrometry (obsd [M + Na]^+^ at *m/z* 289.1417, calcd [M + Na]^+^ 289.1416) as well as ^1^H and ^13^C NMR spectroscopy (Table 2). The IR and mass spectra of **3** were very similar to those of **1** and **2**; however, its UV spectrum displayed an absorption maximum λ_max_ at 271 nm, 31 nm shorter than λ_max_ for **1** and **2**, indicating the presence of a triene moiety in separacene C. Further investigation of the ^1^H, ^13^C, COSY, HSQC, and HMBC NMR spectra revealed that the 3 consecutive double bonds are located between two diols. A careful comparison of **1** and **3** revealed that separacene C (**3**) differs from separacene A (**1**) by the presence of one double bond between the methyl group (C-1) and the diol at C-4 and C-5 (Figure 1). The configurations of the double bonds were established as 2*E*, 6*E*, 8*E*, and 10*E* by their ^1^H-^1^H coupling constants. The modified Mosher method, using MTPA derivatization (**9** and **10**), established the absolute configuration of **3** as 4*R*, 5*R*, 12*S*, and 13*R* (Figure 2).

**Table 2:** NMR data for **3** and **4** in pyridine-*d*_5_.

3							4				
C/H	δ_H_^a^	mult (*J* in Hz)		δ_C_^b^				δ_H_^c^	mult (*J* in Hz)	δ_C_^d^	
**1**	1.67	d (5.0)		18.7	CH_3_			1.66	d (5.0)	18.7	CH_3_
**2**	5.98	m		133.2	CH			5.98	m	133.6	CH
**3**	5.96	dd (15.5, 5.0)		127.4	CH			5.95	dd (15.5, 5.5)	127.8	CH
**4**	4.48	dd (10.5, 5.0)		77.3	CH			4.48	dd (10.5, 5.5)	77.0	CH
**5**	4.58	dd (10.5, 6.0)		76.6	CH			4.58	dd (10.5, 6.0)	76.5	CH
**6**	6.23	dd (15.5, 6.0)		135.8	CH			6.23	dd (15.0, 6.0)	132.0	CH
**7**	6.75	dd (15.5, 10.5)		131.7	CH			6.76	dd (15.0, 11.5)	136.0	CH
**8**	6.42	dd (15.0, 10.5)		133.1	CH			6.42	dd (15.0, 11.5)	133.2	CH
**9**	6.44	dd (15.0, 10.0)		133.0	CH			6.40	dd (15.0, 11.0)	133.0	CH
**10**	6.79	dd (15.5, 10.0)		131.7	CH			6.78	dd (15.5, 11.0)	131.7	CH
**11**	6.35	dd (15.5, 6.0)		136.3	CH			6.26	dd (15.5, 6.0)	136.3	CH
**12**	4.67	dd (11.0, 6.0)		76.5	CH			4.60	dd (10.0, 6.0)	76.6	CH
**13**	4.62	dd (11.0, 5.5)		76.9	CH			4.56	dd (10.0, 5.5)	77.1	CH
**14**	6.48	ddd (17.0, 10.5, 5.5)		140.8	CH			6.36	ddd (17.5, 10.0, 5.5)	140.3	CH
**15**	5.68	dd (17.0, 1.0)		115.6	CH_2_			5.70	dd (17.5, 1.5)	115.9	CH_2_
	5.30	dd (10.5, 1.0)						5.30	dd (10.5, 1.5)		

^a,c^ 600 MHz; ^b,d^ 125 MHz.

Separacene D (**4**) was obtained as a white powder with a molecular formula of C_15_H_22_O_4_, as determined by HR-FAB mass spectrometry (obsd [M + Na]^+^ at *m/z* 289.1426, calcd 289.1416) as well as ^1^H and ^13^C NMR spectroscopy (Table 2). Careful analysis of the ^1^H, ^13^C, COSY, HSQC, and HMBC NMR spectra revealed that separacene D (**4**) has a planar structure identical to **3**, suggesting that separacene D (**4**) is an isomer of **3**. We confirmed the planar structure of **4** by 1D and 2D NMR (Figure 1) and performed MTPA derivatization. After obtaining the tetra-*S*- and -*R*-MTPA esters (**11** and **12**), we analyzed the Δδ*_S-R_* values of **11** and **12** and established the absolute configurations of the four chiral oxygen-bearing carbons as 4*R*, 5*R*, 12*R*, and 13*R* (Figure 2).

Separacenes A–D (**1**–**4**) are novel, linear polyene polyols bearing tetraene or triene units flanked by two diol moieties and a terminal olefin. Although the structures of the separacenes are relatively simple, no comparable natural or synthetic compounds have been reported. The most similar compound reported in the literature is 4,6-decadidene-3,3,9-triol from the fungus *Deuteromycetes* sp. [12]. However, this compound does not share any characteristic features of **1**–**4** such as the tetraene or triene moieties, two diols, and a terminal olefin. Thus, separacenes constitute a novel chemotype.

### 2.2. Bioactivities of Separacenes

The antimicrobial activity of the separacenes was evaluated against diverse pathogenic bacterial strains such as *Staphylococcus aureus* ATCC 6538p, *Bacillus subtilis* ATCC 6633, *Kocuria rhizophila* NBRC 12708, *Salmonella enterica* ATCC 14028, *Proteus hauseri* NBRC 3851, and *Escherichia coli* ATCC 35270. Ampicillin was used as a positive control. Separacene A (**1**) displayed weak antibacterial activity against *B. subtilis* ATCC 6633 and *P. hauseri* NBRC 3851, with MIC values of 50 µg/mL and 100 µg/mL, respectively. However, separacene B–D (**2**–**4**) did not display significant inhibitory activity against the tested bacteria. The separacenes did not exhibit any remarkable activity in antifungal assays against *Candida albicans* ATCC 10231, *Aspergillus fumigatus* HIC 6094, *Trichophyton rubrum* NBRC 9185, and *T. mentagrophytes* IFM 40996. Despite the lack of activity against the pathogenic fungi, separacene A displayed weak inhibitory activity against *C. albicans* isocitrate lyase (ICL), an enzyme that plays an important role in the pathogenicity of *C. albicans* [13], with an IC_50_ value of 45.7 µg/mL (172 µM). Separacenes B–D did not display significant activity in the ICL bioassay. Separacene A displayed weak cytotoxicity against the colon cell line HCT116 and the lung cancer cell line A549, with IC_50_ values of 14.0 µg/mL (52.7 µM) and 37.6 µg/mL (141 µM), respectively. However, separacenes B–D did not display remarkable inhibitory activity against either cancer cell line. 

## 3. Experimental Section

### 3.1. General Experimental Procedures

Optical rotation was measured with a Jasco P-1020 polarimeter with a 1-cm cell. IR spectra were acquired in a Thermo N1COLET iS10 spectrometer. UV spectra were obtained with a Perkin Elmer Lambda 35 UV/VIS spectrometer. Electrospray ionization (ESI) low-resolution LC/MS data were recorded on an Agilent Technologies 6130 Quadrupole mass spectrometer coupled with an Agilent Technologies 1200 series HPLC using a reversed-phase C_18_ column (Phenomenex Luna, 100 × 4.6 mm). High-resolution fast-atom bombardment (HR-FAB) mass spectra were collected with a Jeol JMS-600W high resolution mass spectrometer at NCIRF (National Center for Inter-University Research Facilities). ^1^H, ^13^C, and 2D NMR spectra were obtained on a Bruker Avance 600 MHz spectrometer at NCIRF (National Center for Inter-University Research Facilities) and a 900 MHz NMR spectrometer at KBSI (Korea Basic Science Institute at Ochang).

### 3.2. Isolation of Bacteria, Cultivation, and Extraction

A sediment sample was collected from the southern area of Jeju Island in a 40-mL sterilized plastic tube. The sample (1 g) was diluted in 24 mL sterilized artificial seawater (for 1/6 dilution) and vortexed. The mixture was spread on Actinomycete Isolation Agar, Chitin-based Agar, A4 medium (1 L seawater, 18 g agar, 100 mg/L cycloheximide), and A5 medium (750 mL seawater, 250 mL distilled water, 18 g agar, 100 mg/L cycloheximide). SNJ210 was isolated on A5 media. The analysis of 16S rDNA sequence (Figure S21 in the supplementary data) revealed that SNJ210 is most similar to *Streptomyces sundarbansensis* MS1/7T. The strain was deposited in the National Center for Biotechnology Information, United States, with the accession number of KF318310. The strain SNJ210 was cultivated in 50 mL YEME media (4 g yeast extract, 10 g malt extract, and 4 g glucose in 1 L artificial seawater) in a 125 mL Erlenmeyer flask. After cultivation for 3 days on a rotary shaker at 200 rpm at 30 °C, 10 mL samples of the culture were inoculated in 1 L YPM medium in 2.8 L Fernbach flasks (12 ea × 1 L, total volume 12 L). The large culture was incubated under the same conditions used for the seed culture. After 2 days, the whole culture (12 L) was extracted twice with 18 L ethyl acetate. The ethyl acetate layer was separated and dried over anhydrous sodium sulfate. The ethyl acetate extract was concentrated *in vacuo* to yield 1.5 g of dried material. This procedure was repeated 20 times (240 L culture, the total amount of extract: 30 g) to obtain sufficient quantities of the separacenes for structure elucidation and bioassays.

### 3.3. Isolation of Separacenes A–D

The crude extract was absorbed on celite, loaded on a 2 g Sep-Pak C_18_ cartridge, and fractionated with 20 mL each of 20%, 40%, 60%, 80%, and 100% MeOH in water and 1:1 MeOH/dichloromethane. Separacenes A–D (**1**–**4**) were found in the 20% and 40% MeOH/water fractions. To further purify **1**–**4**, multiple chromatography steps were employed. First, the fractions bearing **1**–**4** were subjected to reversed-phase HPLC [Kromasil C_18_ (2): 250 × 10 mm, 5 µm] under the isocratic conditions in 2:8 acetonitrile/water (UV 280 nm detection, flow rate: 2 mL/min). A total of 4 peaks at the retention times 19 min, 20 min, 23 min, and 25 min were collected. The collected materials were further purified by passage through a cyano HPLC column (YMC CN: 250 × 10 mm, 5 µm) with a gradient solvent system (30% MeOH/water to 50% MeOH/water over 40 min, UV 280 nm detection, flow rate: 2 mL/min). Relatively pure separacenes A–D (**1**–**4**) eluted at 13 min, 11 min, 12 min, and 13.5 min, respectively. In the final purification step, normal-phase HPLC was employed (YMC silica: 250 × 4.6 mm, 5 µm, UV 280 nm detection, flow rate: 1 mL/min). Finally, separacene A (**1**) (11 mg), separacene B (**2**) (3 mg), separacene C (**3**) (5 mg), and separacene D (**4**) (8 mg) were isolated as pure compounds at retention times at 9 min, 8 min, 11 min, and 8.5 min, respectively.

#### 3.3.1. Separacene A (**1**)

[α]_D_ −15 (c 0.075, MeOH); UV (MeOH) λ_max_ (log ε) 302 (4.21) nm; IR (neat) ν_max_ 3370, 2968, 2923, 1647, 1595 cm^−1^; for ^1^H and ^13^C NMR data, see Table 1; HRFABMS *m/z* 289.1417 [M + Na]^+^ (calcd for C_15_H_22_O_4_Na 289.1416).

#### 3.3.2. Separacene B (**2**)

[α]_D_ −12, (c 0.05, MeOH); UV (MeOH) λ_max_ (log ε) 302 (4.13) nm; IR (neat) ν_max_ 3360, 2924, 2853, 1659, 1611 cm^−1^; for ^1^H and ^13^C NMR data, see Table 1; HRFABMS *m/z* 289.1410 [M + Na]^+^ (calcd for C_15_H_22_O_4_Na 289.1416).

#### 3.3.3. Separacene C (**3**)

[α]_D_ −4, (c 0.05, MeOH); UV (MeOH) λ_max_ (log ε) 270 (4.67) nm; IR (neat) ν_max_ 3370, 2958, 2923, 1652, 1596 cm^−1^; for ^1^H and ^13^C NMR data, see Table 2; HRFABMS *m/z* 289.1417 [M + Na]^+^ (calcd for C_15_H_22_O_4_Na 289.1416).

#### 3.3.4. Separacene D (**4**)

[α]_D_ −2, (c 0.05, MeOH); UV (MeOH) λ_max_ (log ε) 270 (4.82) nm; IR (neat) ν_max_ 3360, 2957, 2923, 1658, 1633 cm^−1^; for ^1^H and ^13^C NMR data, see Table 2; HRFABMS *m/z* 289.1426 [M + Na]^+^ (calcd for C_15_H_22_O_4_Na 289.1416).

### 3.4. MTPA Esterification of Separacenes A–D

Separacenes A–D (**1**–**4**) were prepared in eight 40 mL vials (two 1 mg samples for each compound) and were dried completely under high vacuum for 8 h. After adding catalytic amounts of crystalline dimethylaminopyridine (DMAP) to each reaction vial, freshly distilled anhydrous pyridine (1 mL) was added under argon gas. The reaction mixtures were stirred at room temperature for 15 min. After 15 min, *R*- and *S*-α-methoxy trifluoromethyl-phenylacetic acid (MTPA) chloride (30 µL) were separately added. The reactions were quenched by adding 50 µL of MeOH in 1 h. The reaction products were purified on a reversed-phase C_18_ column [Kromasil C_18_ (2): 250 × 10 mm, 5 μm] with a gradient of 40% to 100% aqueous acetonitrile. Tetra-*S*- and -*R*-MTPA esters (**5** and **6**) of separacene A (**1**) eluted at 42.5 and 41.2 min, respectively. Tetra-*S*- and -*R*-MTPA esters (**7** and **8**) of separacene B (**2**) were isolated at 40.5 and 40.0 min. Tetra-*S*- and -*R*-MTPA esters (**9** and **10**) of separacene C (**3**) were obtained at 39.0 and 38.5 min. Tetra-*S*- and -*R*-MTPA esters (**11** and **12**) of separacene D (**4**) were purified at 43.0 min and 42.0 min. The Δδ_S-R_ values around the stereogenic centers of the MTPA esters were assigned by ^1^H, ^1^H-^1^H COSY, HSQC, and HMBC NMR spectra.

#### 3.4.1. Tetra *S*-MTPA Ester (**5**) of Separacene A (**1**)

^1^H NMR (600 MHz, pyridine*-d*_5_) δ 7.76–7.73 (m, 8H), 7.47–7.43 (m, 12H), 6.64 (dd, *J* = 15.5, 10.5, 1H), 6.61 (dd, *J* = 15.5, 10.5, 1H), 6.39–6.32 (m, 4H), 6.13 (dd, *J* = 8.0, 4.0, 1H), 6.05 (m, 1H). 5.97 (m, 1H), 5.93 (ddd, *J* = 17.5, 10.5, 7.0, 1H), 5.85 (dd, *J* = 15.5, 8.0, 1H), 5.79 (dd, *J* = 15.5, 8.0, 1H), 5.58 (m, 1H), 5.52 (dd, *J* = 17.5, 1.0, 1H), 5.35 (dd, *J* = 10.5, 1.0, 1H), 3.58-3.56 (m, 12H), 1.38 (dd, *J* = 6.5, 1.0, 3H). The molecular formula of **5** was confirmed as C_55_H_50_O_12_F_12_ ([M + Na]^+^ at *m/z* 1153).

#### 3.4.2. Tetra-*R*-MTPA Ester (**6**) of Separacene A (**1**)

^1^H NMR (600 MHz, pyridine-*d*_5_) δ 7.75–7.73 (m, 8H), 7.46–7.44 (m, 12H), 6.55 (m, 1H), 6.53 (m, 1H), 6.39–6.37 (m, 4H), 6.18 (dd, *J* = 6.5, 6.5, 1H), 6.11 (dd, *J* = 6.0, 6.0, 1H). 6.01 (dd, *J* = 6.0, 6.0, 1H), 5.96 (ddd, *J* = 17.0, 10.5, 6.5, 1H), 5.87 (dd, *J* = 15.0, 7.0, 1H), 5.84 (dd, *J* = 15.0, 7.5, 1H), 5.65 (m, 1H), 5.46 (dd, *J* = 17.0, 1.0, 1H), 5.34 (dd, *J* = 10.5, 1.0, 1H), 3.62 (s, 3H), 3.61 (s, 3H), 3.60 (s, 3H), 3.59 (s, 3H), 1.37 (d, *J* = 6.5, 3H). The molecular formula of **6** was confirmed as C_55_H_50_O_12_F_12_ (FABMS [M + Na]^+^ at *m/z* 1153).

#### 3.4.3. Tetra-*S*-MTPA ester (**7**) of separacene B (**2**)

^1^H NMR (600 MHz, pyridine-*d*_5_) δ 7.76–7.73 (m, 5H), 7.72–7.69 (m, 3H), 7.48–7.42 (m, 10H), 7.41–7.39 (m, 2H), 6.64 (dd, *J* = 15.0, 9.5, 1H), 6.46 (dd, *J* = 15.5, 9.5, 1H), 6.32–6.37 (m, 4H), 6.13 (dd, *J* = 11.0, 7.0, 1H), 6.05–6.03 (m, 2H). 5.93 (ddd, *J* = 17.5, 10.0, 7.0, 1H), 5.85 (dd, *J* = 15.0, 7.0, 1H), 5.72-5.74 (m, 2H), 5.51 (dd, *J* = 17.5, 1.0, 1H), 5.34 (dd, *J* = 10.0, 1H), 3.59 (s, 3H), 3.58–3.56 (m, 9H), 1.39 (d, *J* = 6.5, 3H). The molecular formula of the **7** was confirmed as C_55_H_50_O_12_F_12_ (FABMS [M + Na]^+^ at *m/z* 1153).

#### 3.4.4. Tetra-*R*-MTPA Ester (**8**) of Separacene B (**2**)

^1^H NMR (600 MHz, pyridine-*d*_5_) δ 7.75–7.72 (m, 6H), 7.71 (m, 2H), 7.46–7.43 (m, 10H), 7.41 (m, 2H), 6.71 (dd, *J* = 15.0, 10.5, 1H), 6.53 (dd, *J* = 15.0, 10.0, 1H), 6.40–6.32 (m, 4H), 6.17 (dd, *J* = 11.0, 7.0, 1H), 6.10 (dd, *J* = 11.0, 4.0, 1H), 6.08 (m, 1H), 5.96 (dd, *J* = 15.0, 10.5, 1H), 5.94 (ddd, *J* = 17.0, 10.5, 4.0, 1H), 5.87 (dd, *J* = 15.0, 7.0, 1H), 5.64 (m, 1H), 5.45 (dd, *J* = 17.0, 1.0, 1H), 5.34 (dd, *J* = 10.5, 1.0, 1H), 3.62 (s, 3H), 3.61 (s, 3H), 3.58 (s, 3H), 3.57 (s, 3H), 1.24 (d, *J* = 6.5, 3H). The molecular formula of **8** was confirmed as C_55_H_50_O_12_F_12_ (FABMS [M + Na]^+^ at *m/z* 1153). 

#### 3.4.5. Tetra-*S*-MTPA Ester (**9**) of Separacene C (**3**)

^1^H NMR (600 MHz, pyridine-*d*_5_) δ 7.77–7.73 (m, 8H), 7.46–7.42 (m, 12H), 6.59 (dd, *J* = 15.5, 10.0 1H), 6.45 (dd, *J* = 15.5, 10.0 1H), 6.23–6.20 (m, 2H), 6.17-6.15 (m, 2H), 6.11 (m, 1H). 6.06–6.04 (m, 2H), 6.00 (m, 1H), 5.86 (dd, *J* = 15.5, 8.0, 1H), 5.75 (dd, *J* = 15.5, 8.0, 1H), 5.62 (d, *J* = 17.0, 1H), 5.58 (m, 1H), 5.42 (d, *J* = 10.5, 1H), 3.55 (s, 3H), 3.53–3.50 (m, 9H), 1.57 (d, *J* = 6.0, 3H). The molecular formula of the **9** was confirmed as C_55_H_50_O_12_F_12_ (FABMS [M + Na]^+^ at *m/z* 1153).

#### 3.4.6. Tetra-*R*-MTPA Ester (**10**) of Separacene C (**3**)

^1^H NMR (600 MHz, pyridine-*d*_5_) δ 7.69–7.63 (8H), 7.38–7.35 (m, 12H), 6.61 (dd, *J* = 15.5, 10.5, 1H), 6.48 (dd, *J* = 15.5, 10.5, 1H), 6.28 (dd, *J* = 15.5, 10.5, 1H), 6.21 (dd, *J* = 15.5, 10.5, 1H), 6.14 (dd, *J* = 8.0, 3.5, 1H). 6.10 (m, 1H), 6.05 (dd, *J* = 12.0, 5.5, 1H), 6.04 (m, 1H), 5.97 (dd, *J* = 15.0, 7.5, 1H), 5.93 (dd, *J* = 15.5, 10.5, 1H), 5.91 (dd, *J* = 15.5, 10.5, 1H), 5.82 (dd, *J* = 15.0, 7.5, 1H), 5.76 (ddd, *J* = 17.5, 10.5, 7.0, 1H), 5.32 (dd, *J* = 17.5, 1.0, 1H), 5.23 (dd, *J* = 10.5, 1.0, 1H), 3.62 (m, 3H), 3.61 (m, 3H), 3.59 (m, 3H), 3.57 (m, 3H), 1.51 (d, *J* = 6.5, 3H). The molecular formula of **10** was confirmed as C_55_H_50_O_12_F_12_ (FABMS [M + Na]^+^ at *m/z* 1153). 

#### 3.4.7. Tetra-*S*-MTPA Ester (**11**) of Separacene D (**4**)

^1^H NMR (600 MHz, pyridine-*d*_5_) δ 7.70–7.66 (m, 8H), 7.39–7.36 (m, 12H), 6.62 (dd, *J* = 15.5, 10.0, 1H), 6.59 (dd, *J* = 15.5, 10.0, 1H), 6.27–6.25 (m, 2H), 6.13 (dd, *J* = 8.0, 4.0, 1H), 6.07–6.05 (m, 2H). 6.02–5.98 (m, 2H), 5.95 (ddd, *J* = 17.0, 10.5, 7.0, 1H), 5.86 (dd, *J* = 15.5, 7.5, 1H), 5.84 (dd, *J* = 15.5, 7.5, 1H), 5.57 (dd, *J* = 15.5, 7.5, 1H), 5.52 (d, *J* = 17.0, 1H), 5.35 (d, *J* = 10.5, 1H), 3.53 (s, 3H), 3.51 (m, 6H), 3.49 (s, 3H), 1.57 (d, *J* = 6.5, 3H). The molecular formula of **11** was confirmed as C_55_H_50_O_12_F_12_ (FABMS [M + Na]^+^ *m/z* 1153). 

#### 3.4.8. Tetra-*R*-MTPA Ester (**12**) of Separacene D (**4**)

^1^H NMR (600 MHz, pyridine*-d*_5_) δ 7.75–7.72 (m, 8H), 7.46–7.42 (m, 12H), 6.54 (m, 1H), 6.50 (m, 1H), 6.29–6.27 (m, 2H), 6.18 (dd, *J* = 6.5, 6.0, 1H), 6.12 (dd, *J* = 11.0, 6.0, 1H). 6.10 (dd, *J* = 11.0, 6.0, 1H), 6.05 (dd, *J* = 7.0, 7.0, 1H), 5.99 (m, 1H), 5.96 (ddd, *J* = 17.0, 10.5, 6.5, 1H), 5.90 (ddd, *J* = 15.0, 10.5, 7.0, 1H), 5.59 (dd, *J* = 7.5, 2.0, 1H), 5.57 (dd, *J* = 7.5, 2.0, 1H), 5.47 (d, *J* = 17.0, 1H), 5.36 (d, *J* = 10.5, 1H), 3.63 (s, 3H), 3.62 (m, 6H), 3.60 (s, 3H), 1.59 (d, *J* = 6.5, 3H). The molecular formula of **12** was confirmed as C_55_H_50_O_12_F_12_ (FABMS [M + Na]^+^ at *m/z* 1153).

### 3.5. Antibacterial Activity Assay

Gram-positive bacteria (*S. aureus* ATCC 6538p, *B. subtilis* ATCC 6633, *K. rhizophila* NBRC 12708) and Gram-negative bacteria (*S. enterica* ATCC 14028, *P. hauseri* NBRC 3851, *E. coli* ATCC 35270) were used for antimicrobial activity tests. Bacteria were grown overnight in Luria Bertani (LB) broth at 37 °C, harvested by centrifugation, and washed twice with sterile distilled water. Stock solutions of separacene A–D (**1**–**4**) were prepared in DMSO. Each stock solution was diluted with m Plate Count Broth (Difco) to give serial 2-fold dilutions in the range of 50 to 0.8 µg/mL. Aliquots (10 µL) of the broth containing approximately 10^5^ colony-forming units (cfu)/mL of the bacteria were added to each well of a 96-well microtiter plate. The plates were incubated for 12 h at 37 °C. The minimum inhibitory concentration (MIC) values were determined as the lowest concentration of test compound that inhibited bacterial growth. Ampicillin was used as a reference compound. 

### 3.6. Antifungal Activity Assay

YPD medium (1% yeast extract, 2% peptone, 2% dextrose) was used to cultivate *C. albicans* ATCC 10231. After incubation for 48 h at 28 °C, yeast cells were harvested by centrifugation and washed twice with sterile distilled water. *A. fumigatus* HIC 6094, *T. rubrum* NBRC 9185, and *T. mentagrophytes* IFM 40996 were plated on potato dextrose agar and incubated for 2 weeks at 28 °C. Spores were harvested and washed twice with sterile distilled water, and resuspended in distilled water to adjust an initial inoculum size of 10^5^ spores/mL. In each well of a 96-well plate, 90 µL of cells (10^4^ cells/mL) was mixed with test compound solutions (separacenes A–D) in 5% DMSO. A culture with DMSO (0.5%) was used as solvent control, and a culture supplemented with amphotericin B was used as a positive control.

### 3.7. Isocitrate Lyase (ICL) Activity Assay

The formation of glyoxylate phenylhydrazone in the presence of phenylhydrazine and isocitrate was measured at 324 nm. The enzyme reaction mixture (1 mL) consisted of 1.27 mM threo-*DS* (+) isocitrate, 3.75 mM MgCl_2_, 4.1 mM phenylhydrazine, 20 mM sodium phosphate buffer (pH 7.0), and 2.5 µg/mL purified *C. albicans* ICL. The reaction was performed at 37 °C for 30 min with and without adding **1**–**4** dissolved in DMSO (final conc., 0.5%). The protein concentration was measured by the method of Bradford with the Bio-Rad protein assay Kit (Bio-Rad, USA) and bovine serum albumin as a standard. 3-Nitropropionic acid, which inhibits ICL with an IC_50_ value of 1.35 µg/mL (11.3 µM), was used as a positive control.

### 3.8. Evaluation of Anti-Proliferative Activity

The effects of separacenes A–D (**1**–**4**) on cell proliferation were evaluated by the sulforhodamine B (SRB) cellular protein-staining method with some modifications. Briefly, A549 (lung cancer) and HCT116 (colon cancer) cells (1 × 10^4^ cells in 190 µL of complete RPMI 1640 medium) were seeded in a 96-well plate with various concentrations of **1**–**4** and incubated at 37 °C in a humidified atmosphere with 5% CO_2_. After 72 h of treatment with separacenes A–D (**1**–**4**), cells were fixed with 10% TCA solution for 1 h, and cellular proteins were stained with 0.4% SRB in 1% acetic acid solution. Stained cells were dissolved in 10 mM Tris buffer (pH 10.0). The effects of **1**–**4** on cell viability were calculated as percentages relative to the solvent-treated control. The IC_50_ values were calculated using nonlinear regression analysis (percent survival *versus* concentration).

## 4. Conclusions

Chemical analyses of marine actinomycetes strains resulted in the discovery of compounds of a novel linear polyene polyol chemotype, separacenes A–D (**1**–**4**). The separacenes are structurally novel and bear a tetraene or triene flanked by two diol moieties and a terminal olefin. Separacene A displayed inhibitory activity against C. albicans isocitrate lyase. The discovery of these new polyene polyols provides additional evidence that the chemical investigation of marine actinomycetes may lead to the identification of significant natural chemical diversity.

## Supplementary Files

Supplementary File 1 Supplementary Materials (PDF, 1144 KB)
