# Supplementary material for: Separacenes A–D, Novel Polyene Polyols from the Marine Actinomycete, Streptomyces sp
Source: Mar Drugs. 2013 Aug 13;11(8):2882–93. doi: 10.3390/md11082882 (PMC3766871; doi:10.3390/md11082882)
Supplement: Supplementary File 1 — Supplementary Materials (PDF, 1144 KB) [file marinedrugs-11-02882-s001.pdf]

Supplementary Materials

Figure S1. ^{1}H NMR spectrum of separacene A (**1**) at 900 MHz in pyridine-d_5.

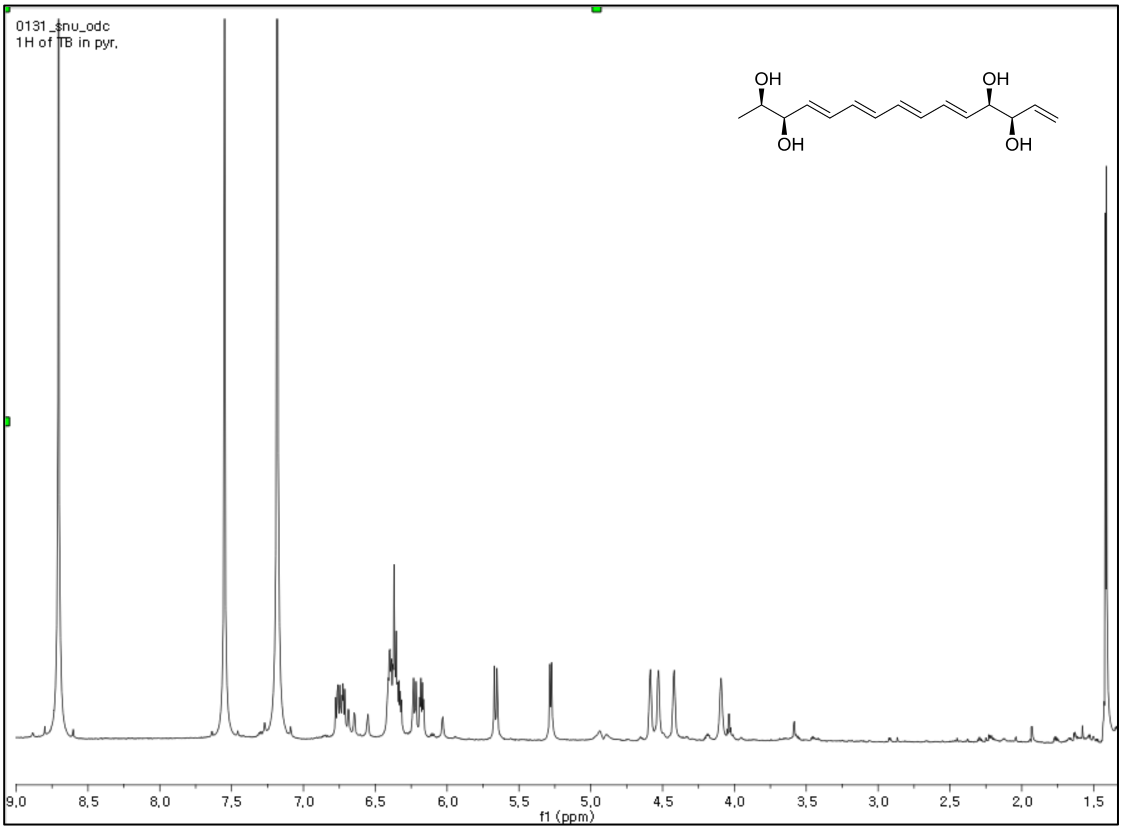

Figure S2. ^{13}C NMR spectrum of separacene A (**1**) at 225 MHz in pyridine-d_5.

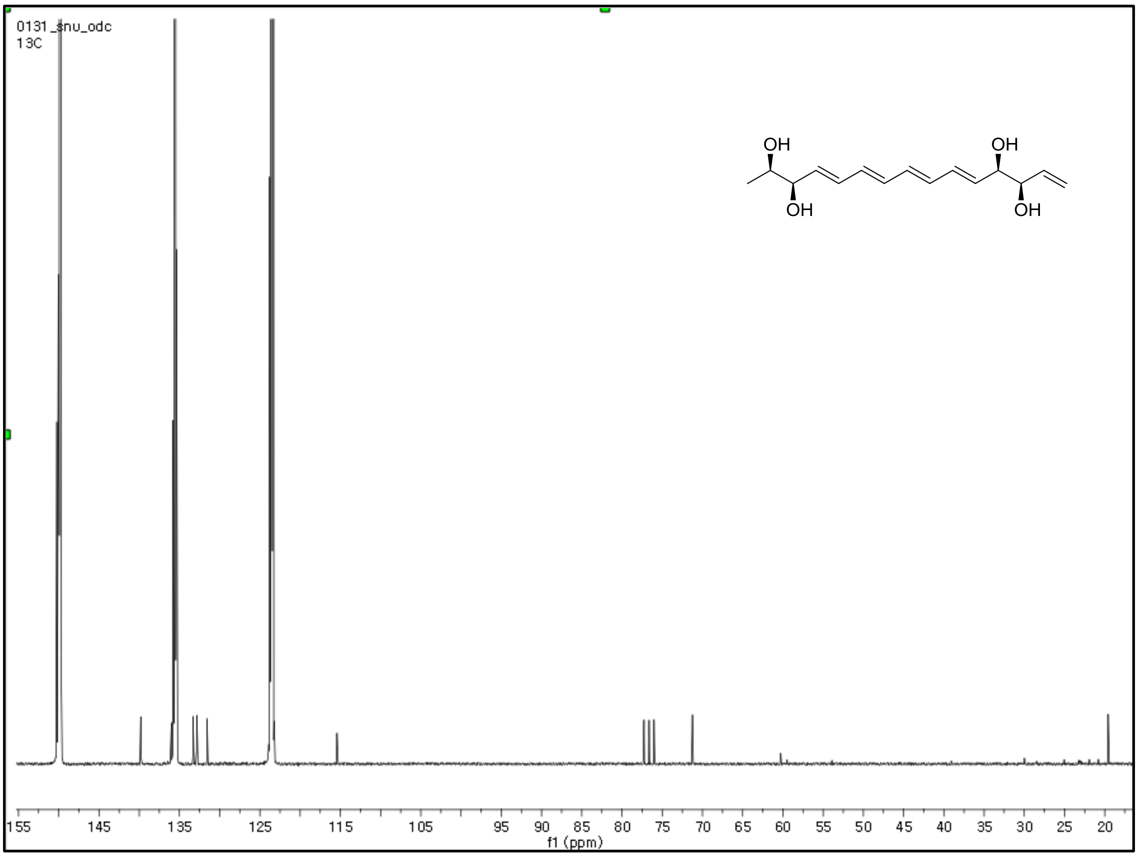

Figure S3. ^{1}H-^{1}H COSY spectrum of separacene A (**1**) at 900 MHz in pyridine-d_5.

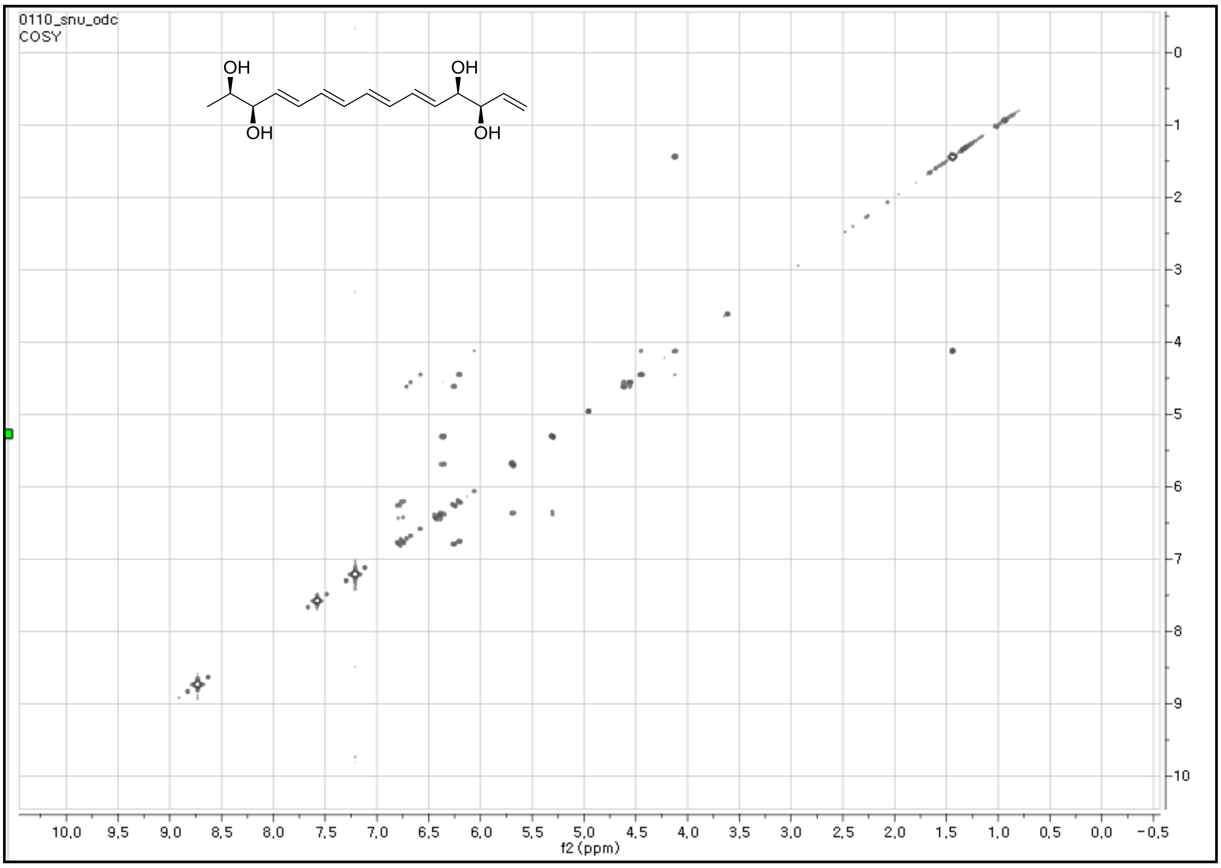

Figure S4. HSQC spectrum of separacene A (**1**) at 900 MHz in pyridine-d_5.

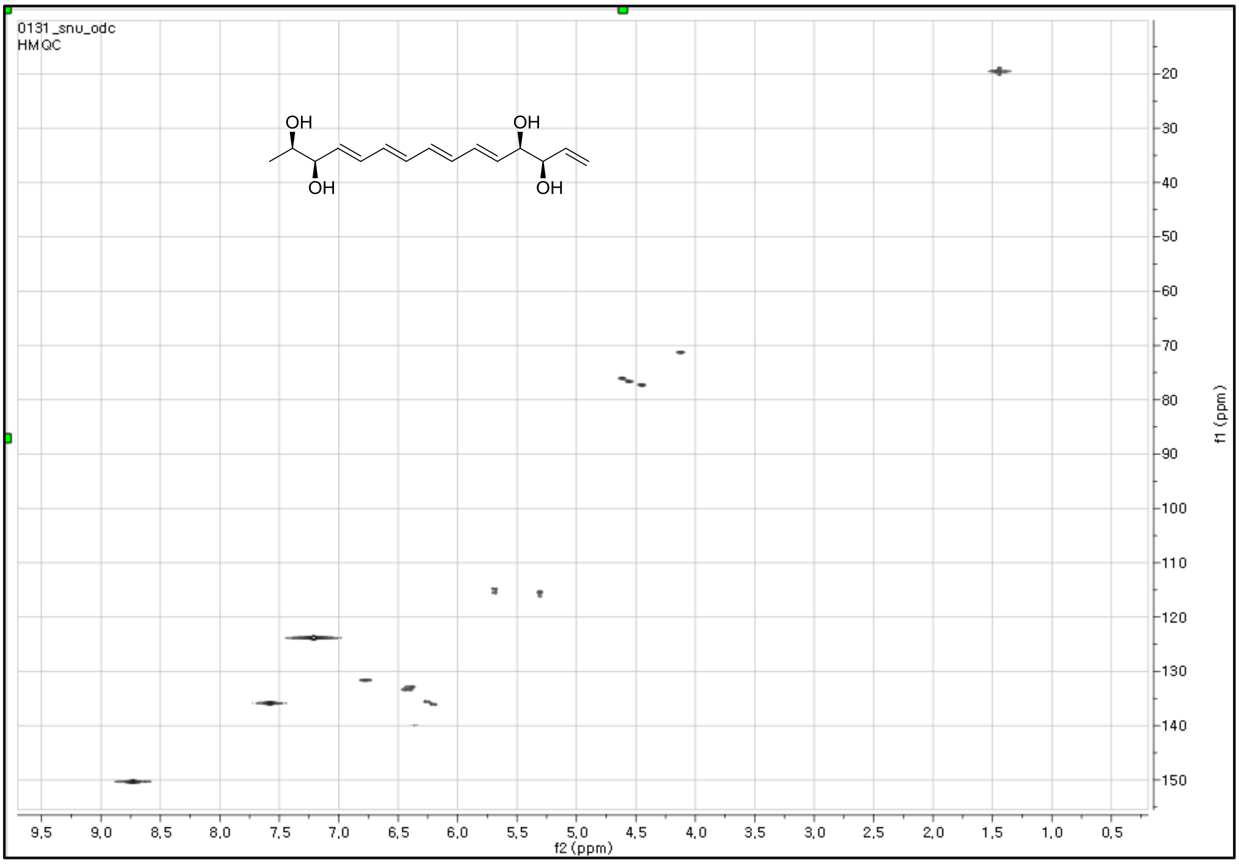

Figure S5. HMBC spectrum of separacene A (**1**) at 900 MHz in pyridine-d_5.

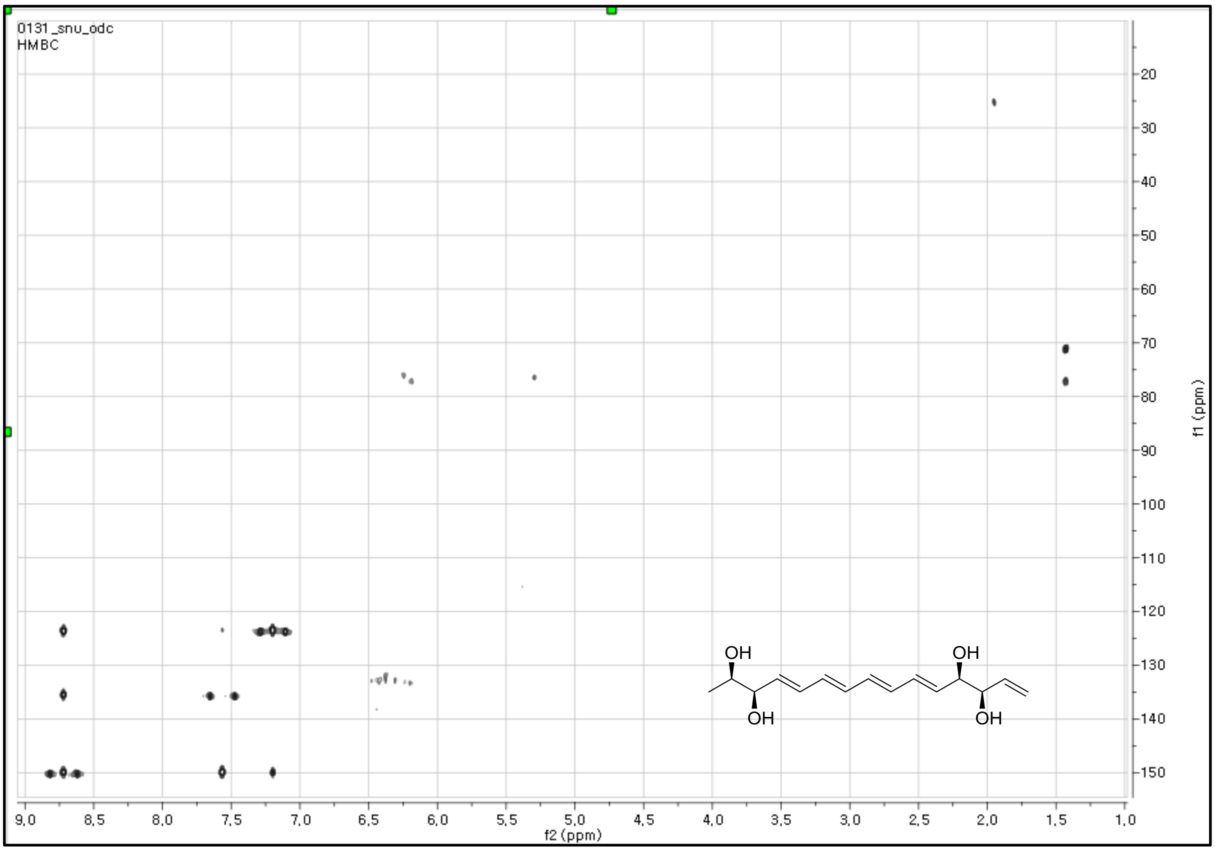

Figure S6. NOESY spectrum of separacene A (**1**) at 900 MHz in pyridine-d_5.

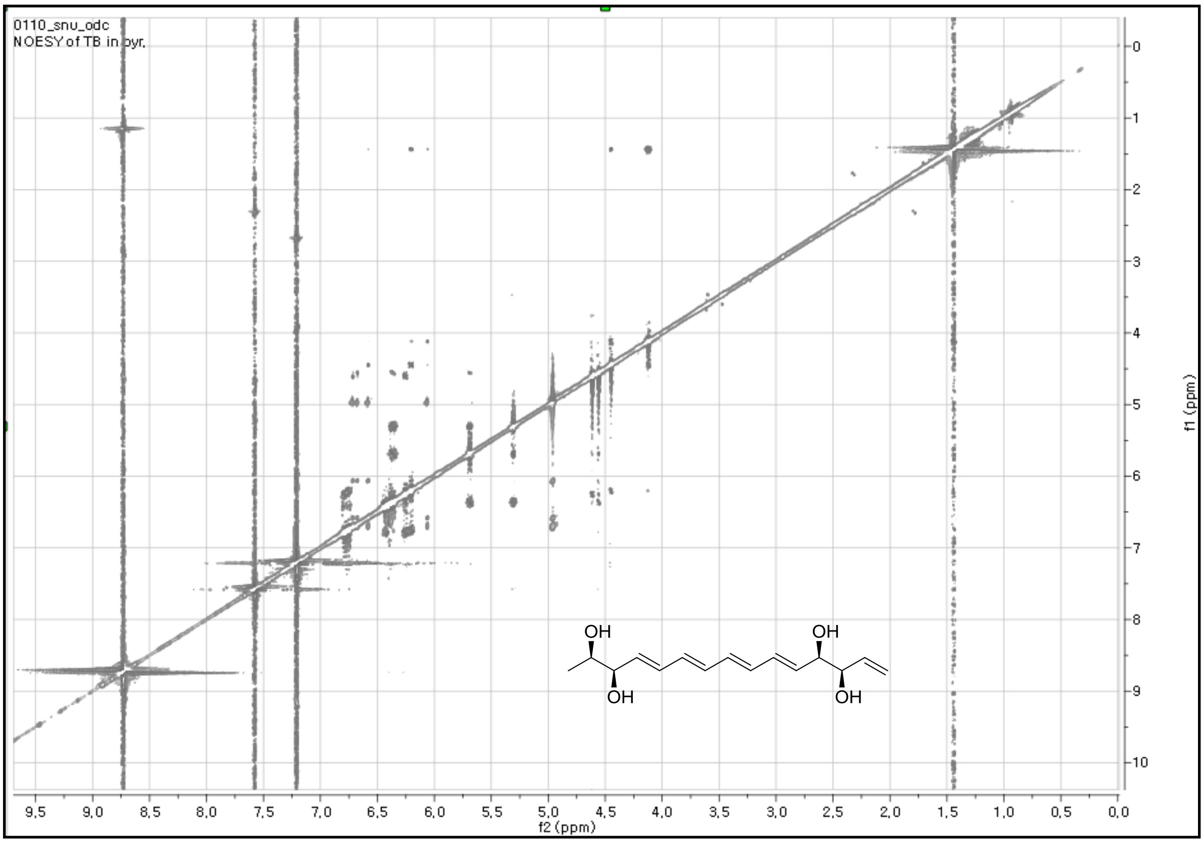

Figure S7. ^{1}H NMR spectrum of separacene B (**2**) at 600 MHz in pyridine-d_5.

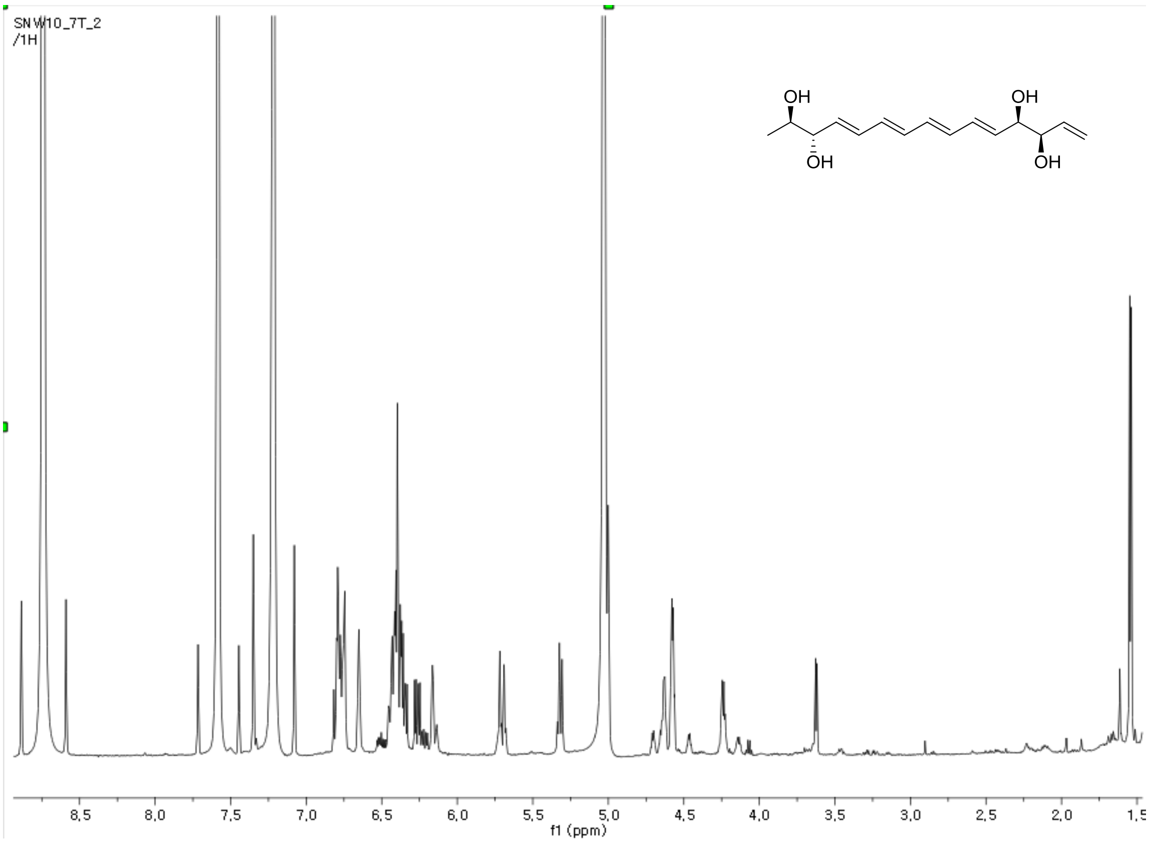

Figure S8. ^{13}C NMR spectrum of separacene B (**2**) at 125 MHz in pyridine-d_5.

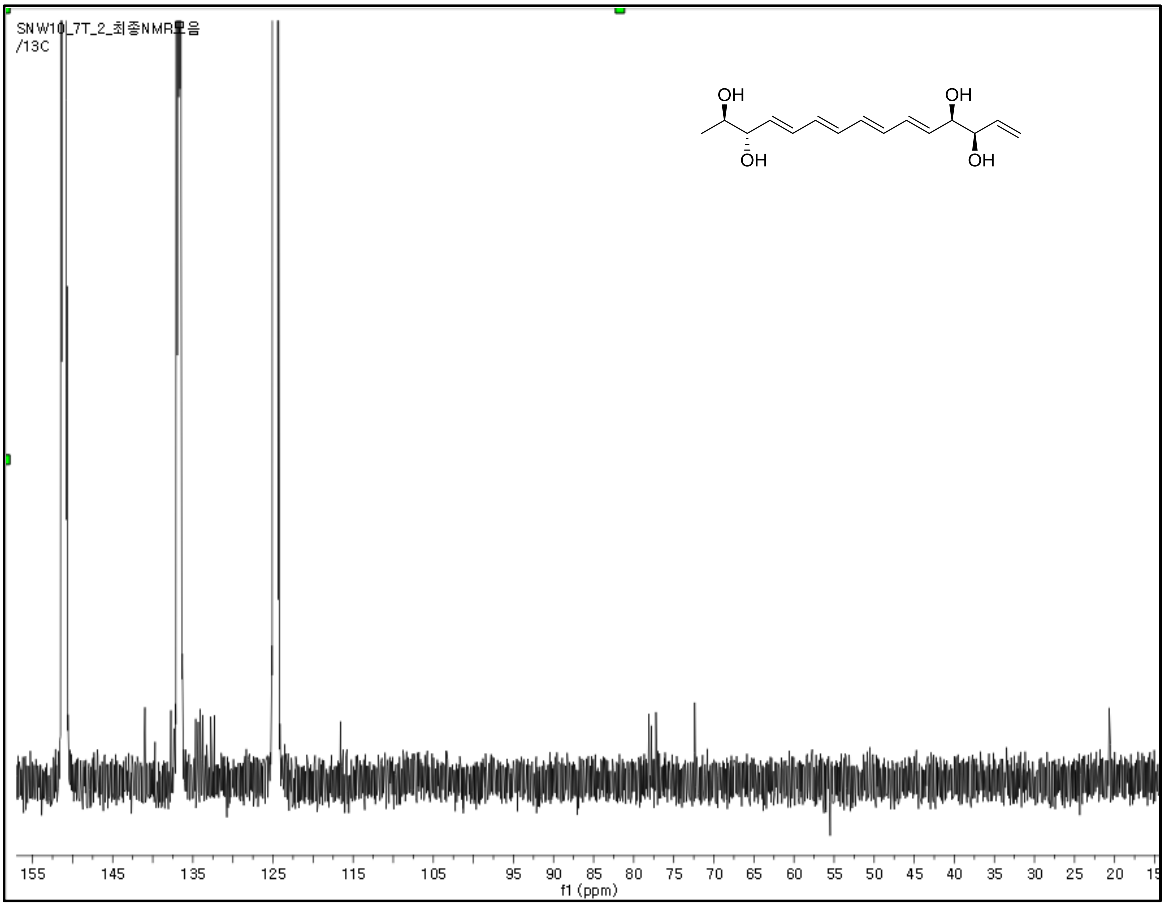

Figure S9. ^{1}H NMR spectrum of separacene C (**3**) at 600 MHz in pyridine-d_5.

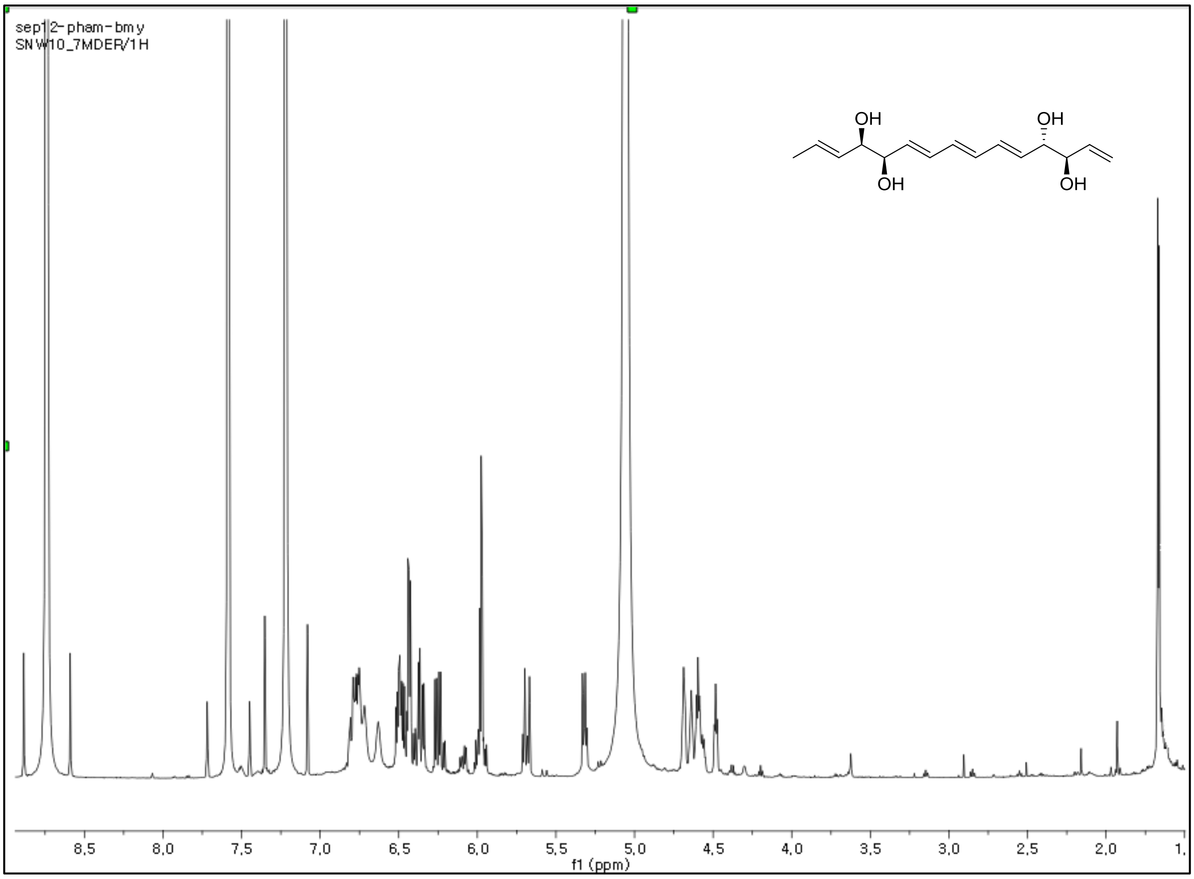

Figure S10. ^{13}C NMR spectrum of separacene C (**3**) at 125 MHz in pyridine-d_5.

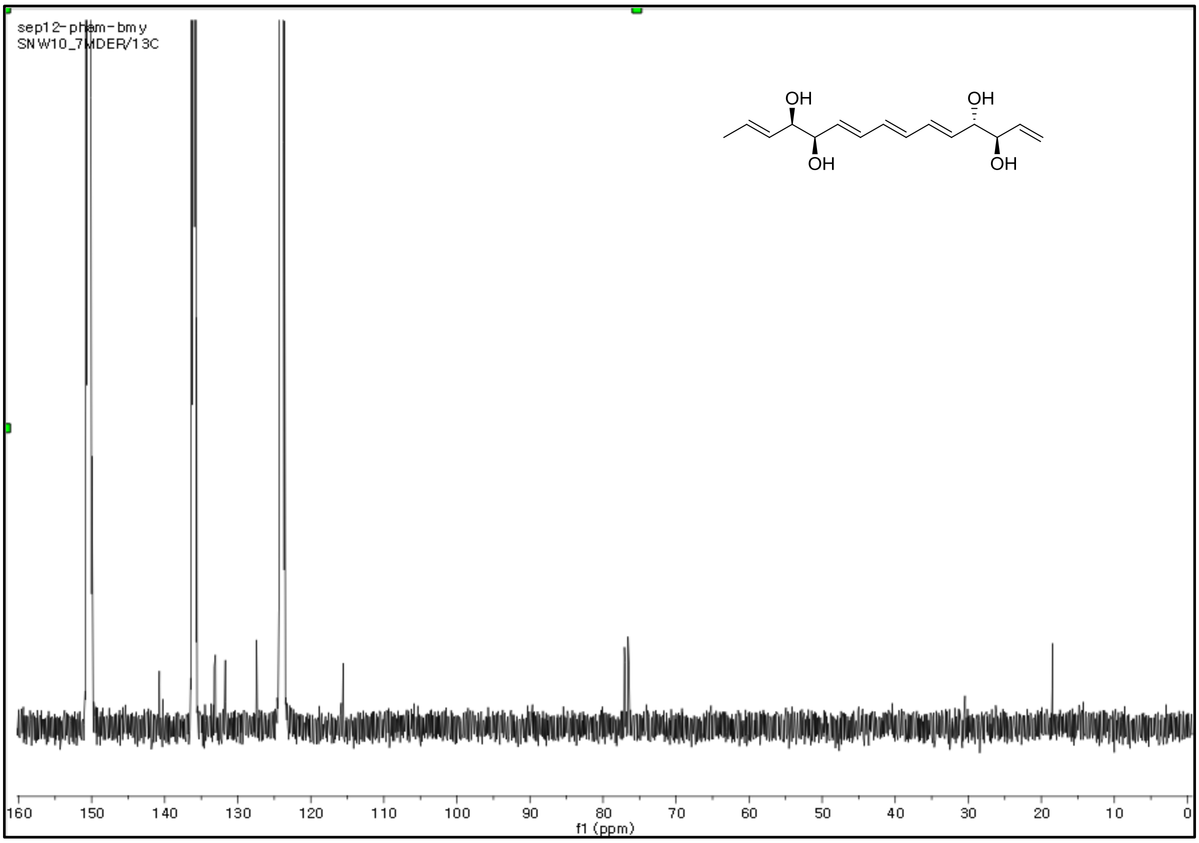

Figure S11. ^{1}H NMR spectrum of separacene D (**4**) at 600 MHz in pyridine-d_5.

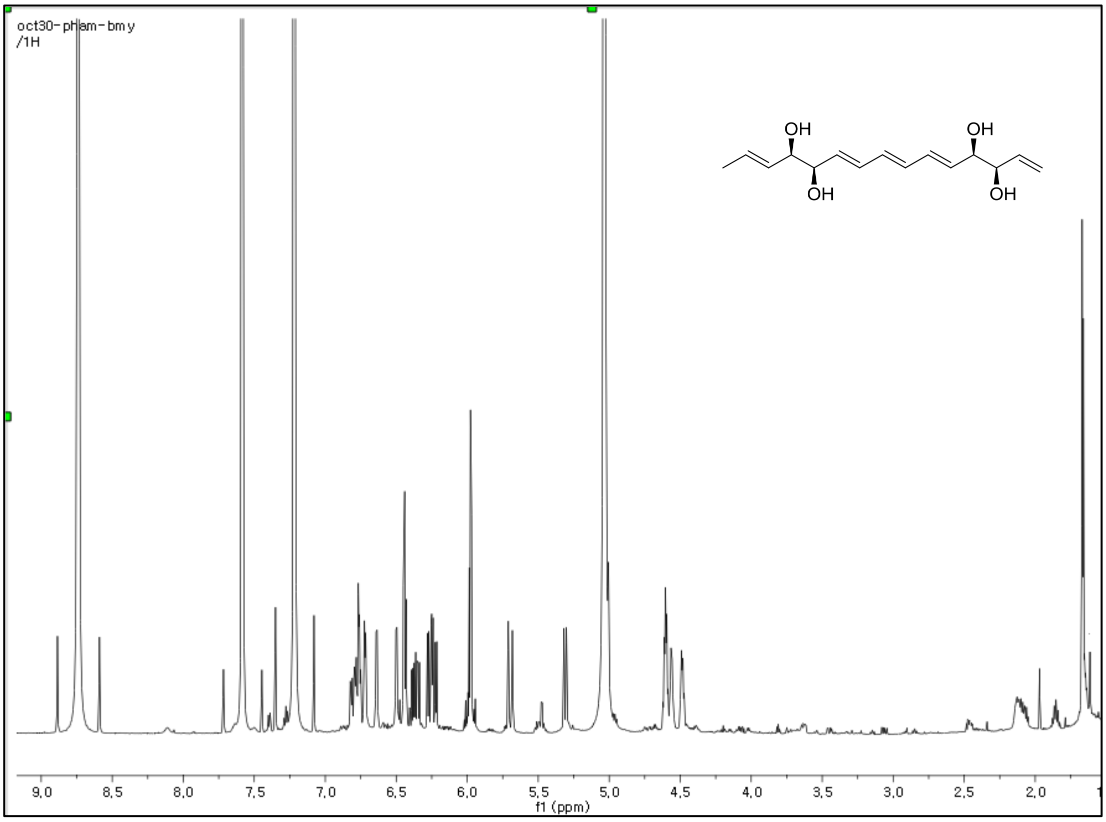

Figure S12. ^{13}C NMR spectrum of separacene D (**4**) at 125 MHz in pyridine-d_5.

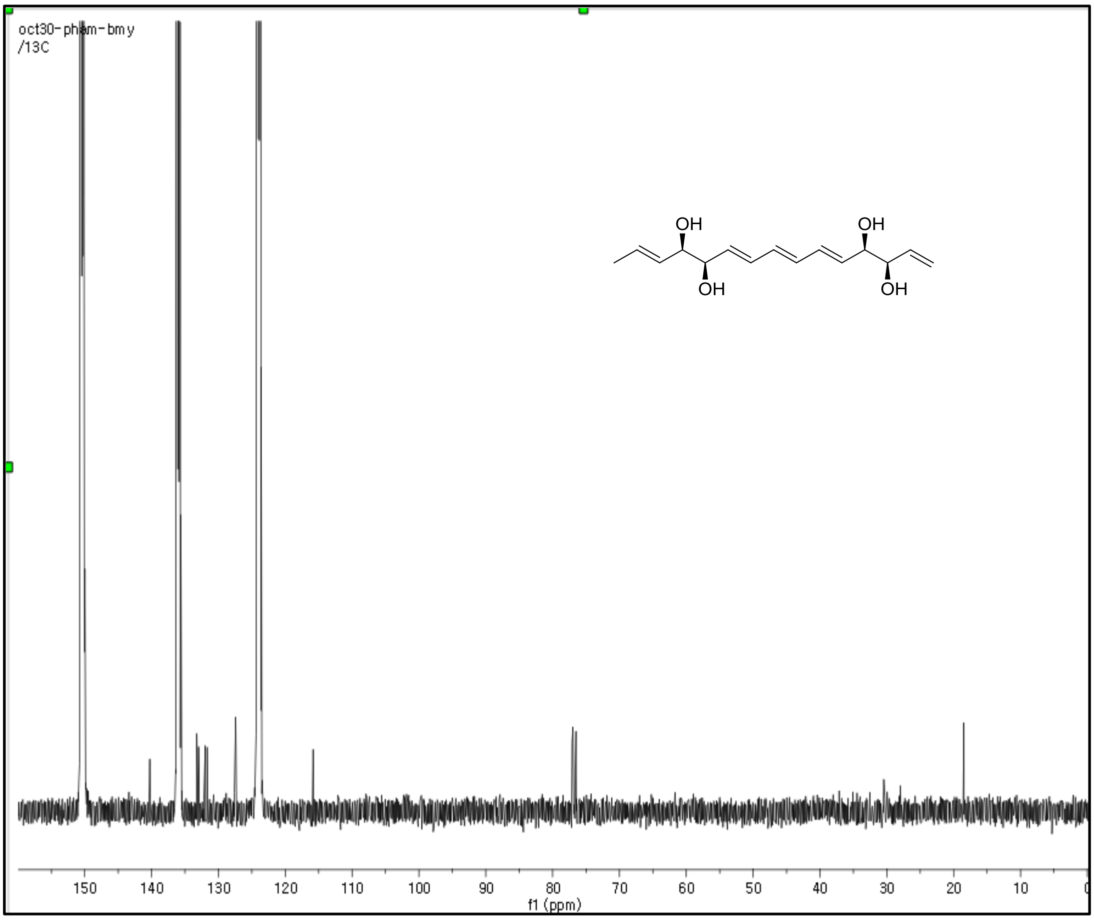

Figure S13. ^{1}H NMR spectrum of *S*-MTPA ester (**5**) for separacene A (**1**) at 600 MHz in pyridine-d_5.

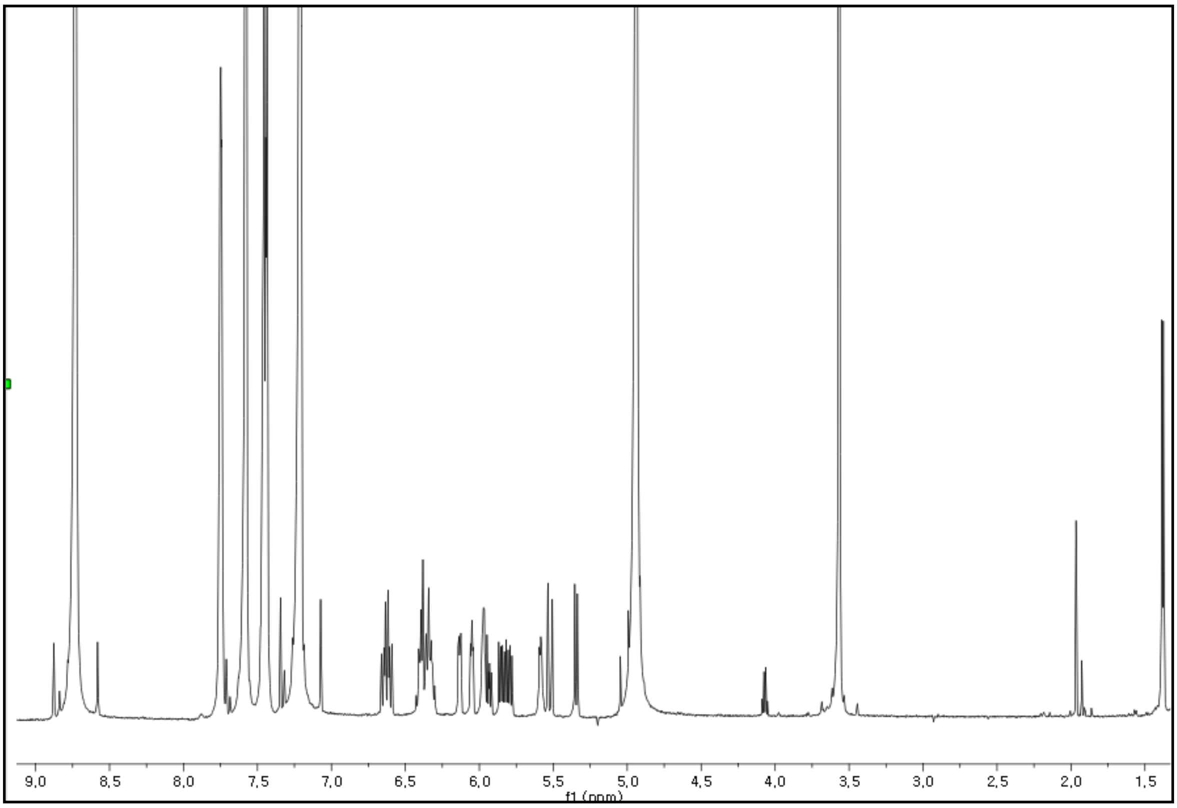

Figure S14. ^{1}H NMR spectrum of *R*-MTPA (**6**) ester for separacene A (**1**) at 600 MHz in pyridine-d_5.

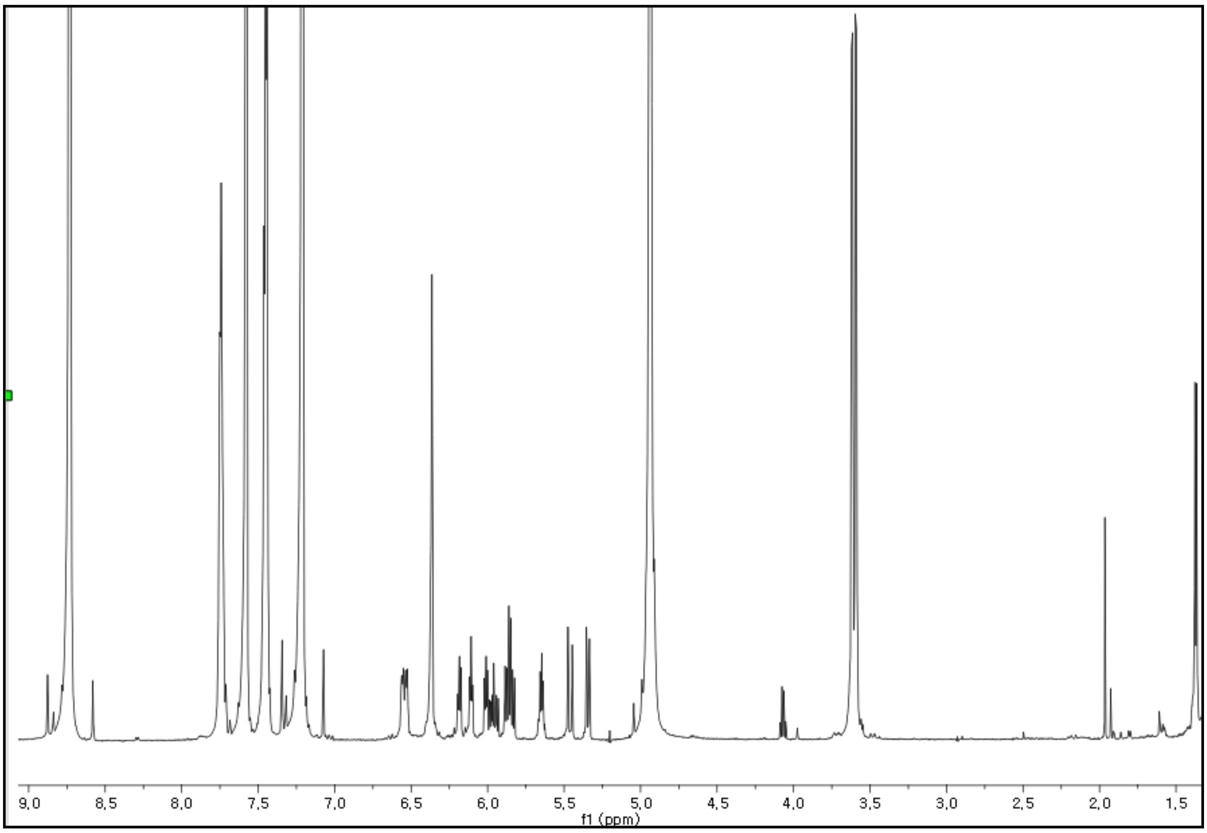

Figure S15. ^{1}H NMR spectrum of *S*-MTPA ester (**7**) for separacene B (**2**) at 600 MHz in pyridine-d_5.

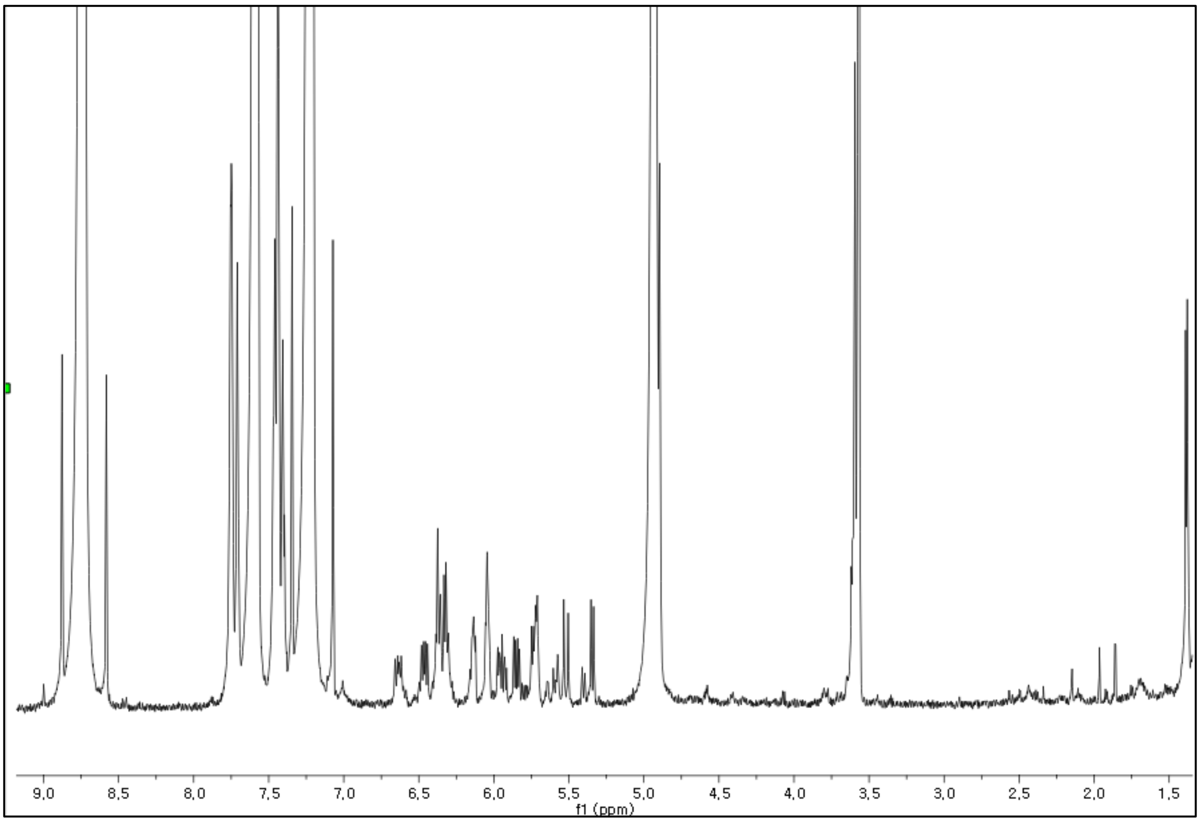

Figure S16. ^{1}H NMR spectrum of *R*-MTPA ester (**8**) for separacene B (**2**) at 600 MHz in pyridine-d_5.

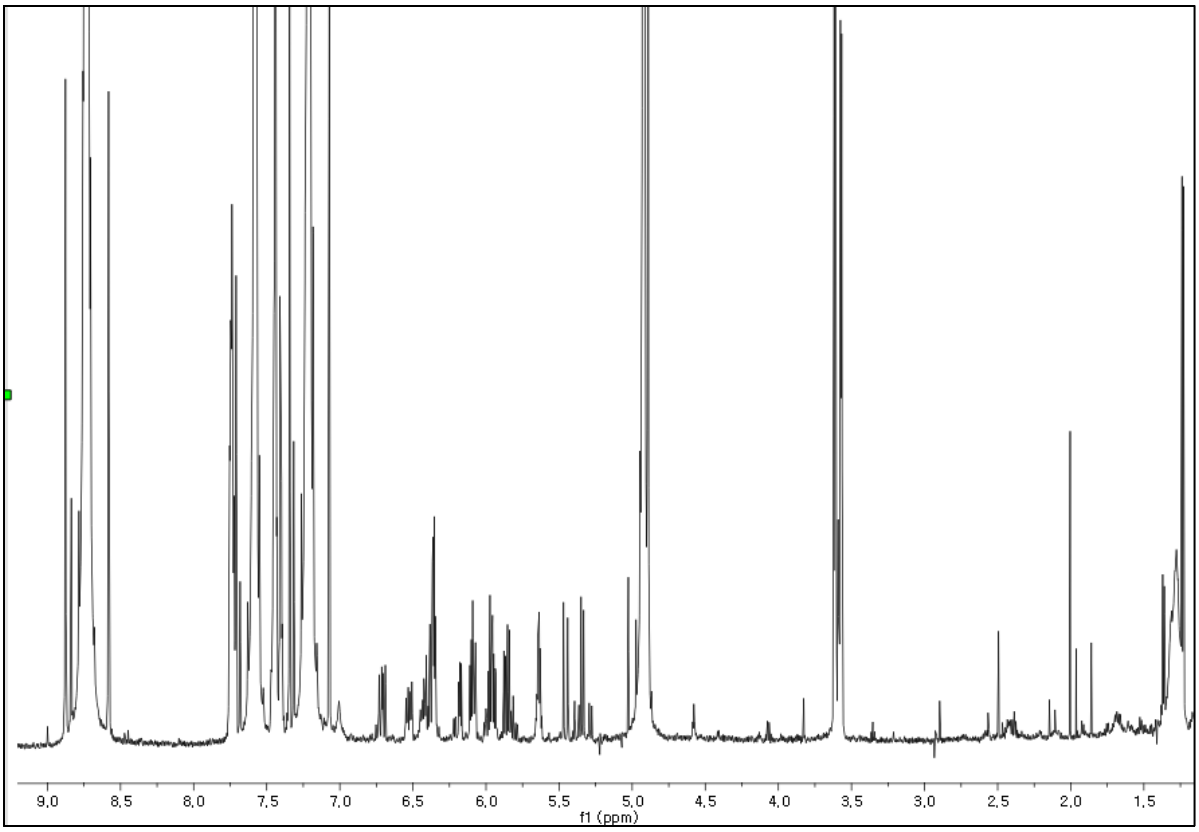

Figure S17. ^{1}H NMR spectrum of *S*-MTPA ester (**9**) for separacene C (**3**) at 600 MHz in pyridine-d_5.

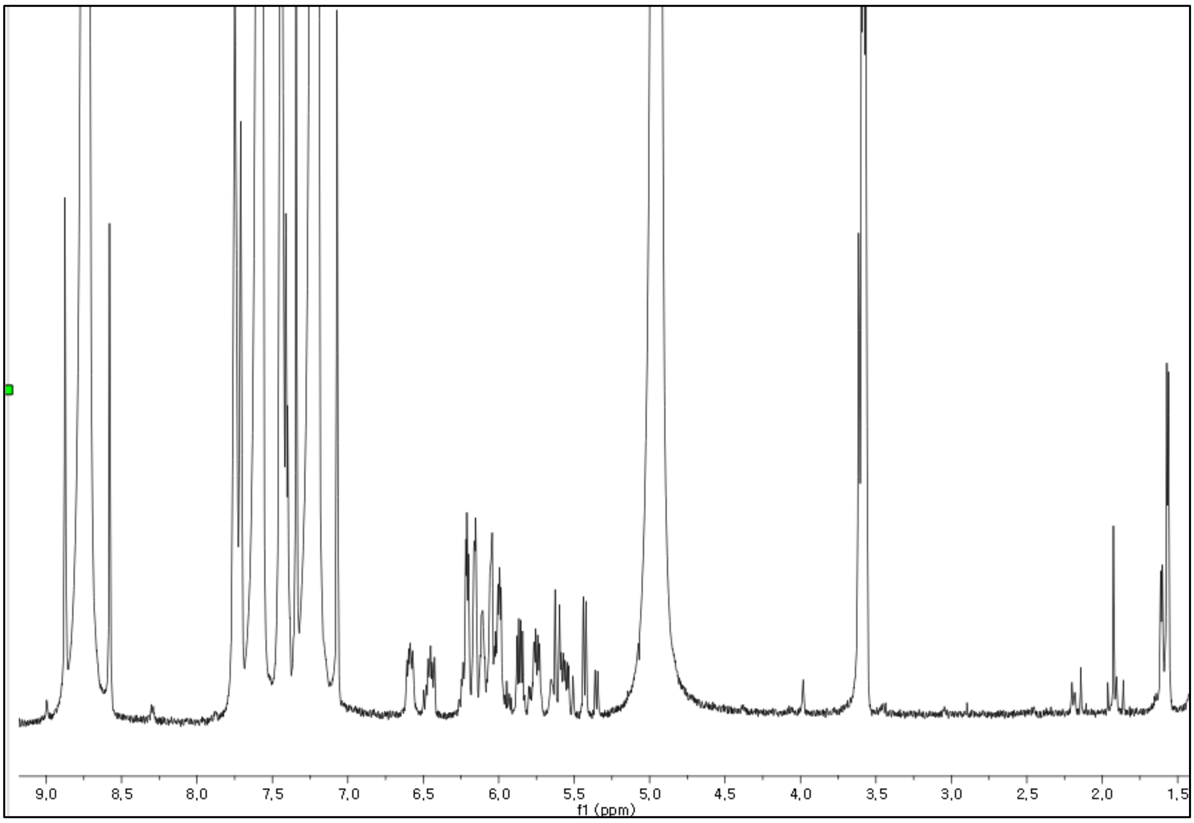

Figure S18. ^{1}H NMR spectrum of *R*-MTPA ester (**10**) for separacene C (**3**) at 600 MHz in pyridine-d_5.

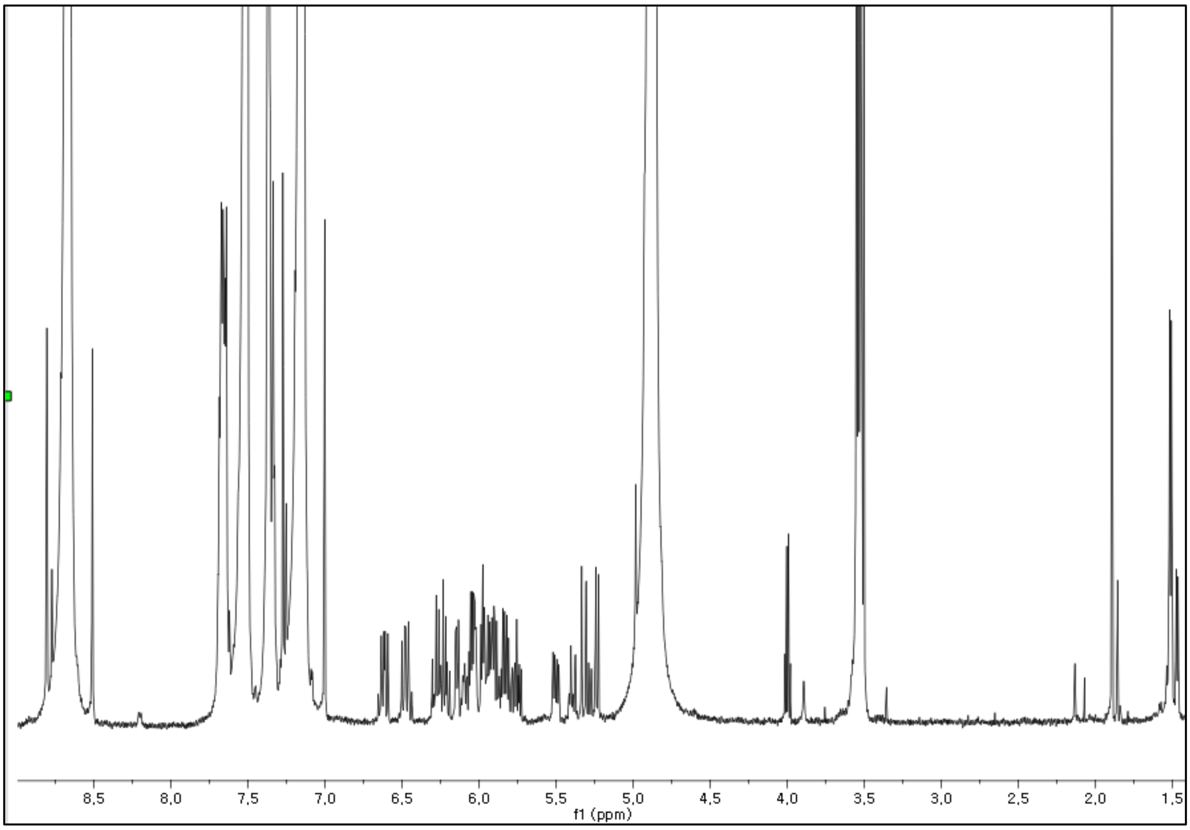

Figure S19. ¹H NMR spectrum of *S*-MTPA ester (**11**) for separacene D (**4**) at 600 MHz in pyridine-d_5.

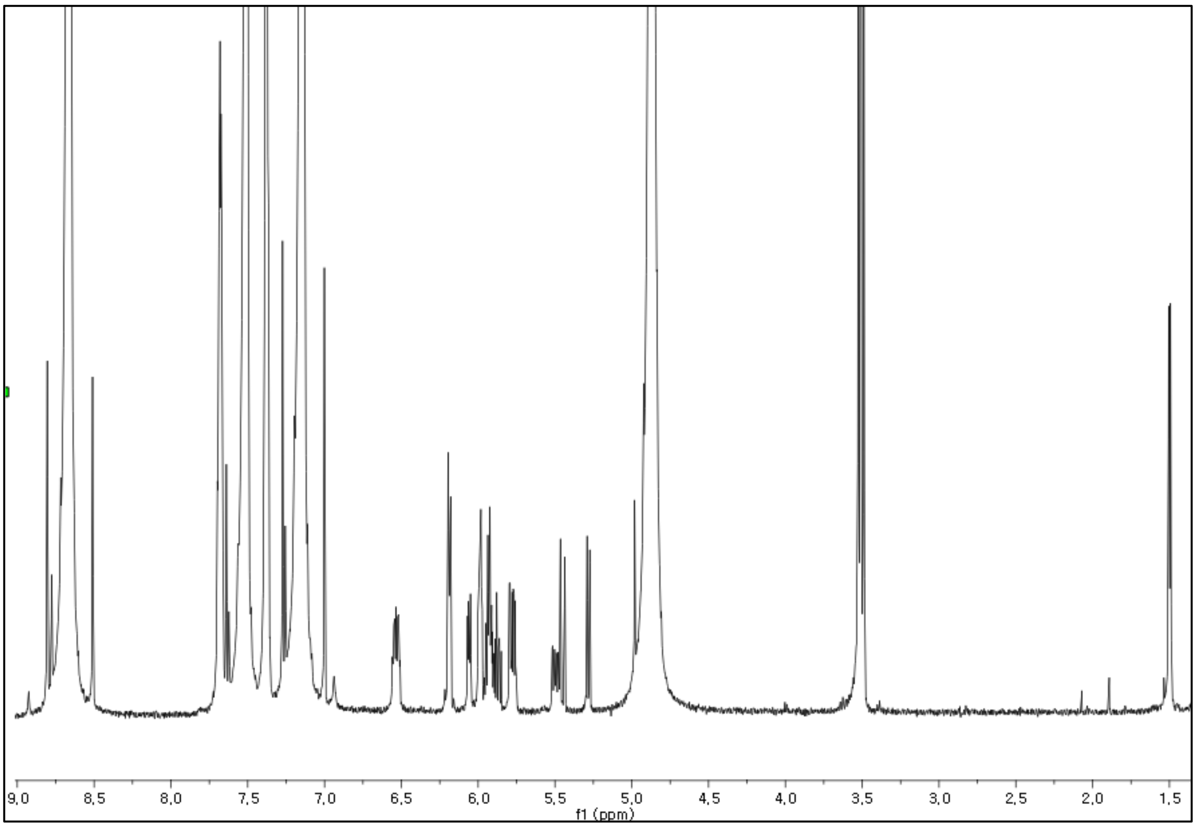

Figure S20. ¹H NMR spectrum of *R*-MTPA ester (**12**) for separacene D (**4**) at 600 MHz in pyridine-d_5.

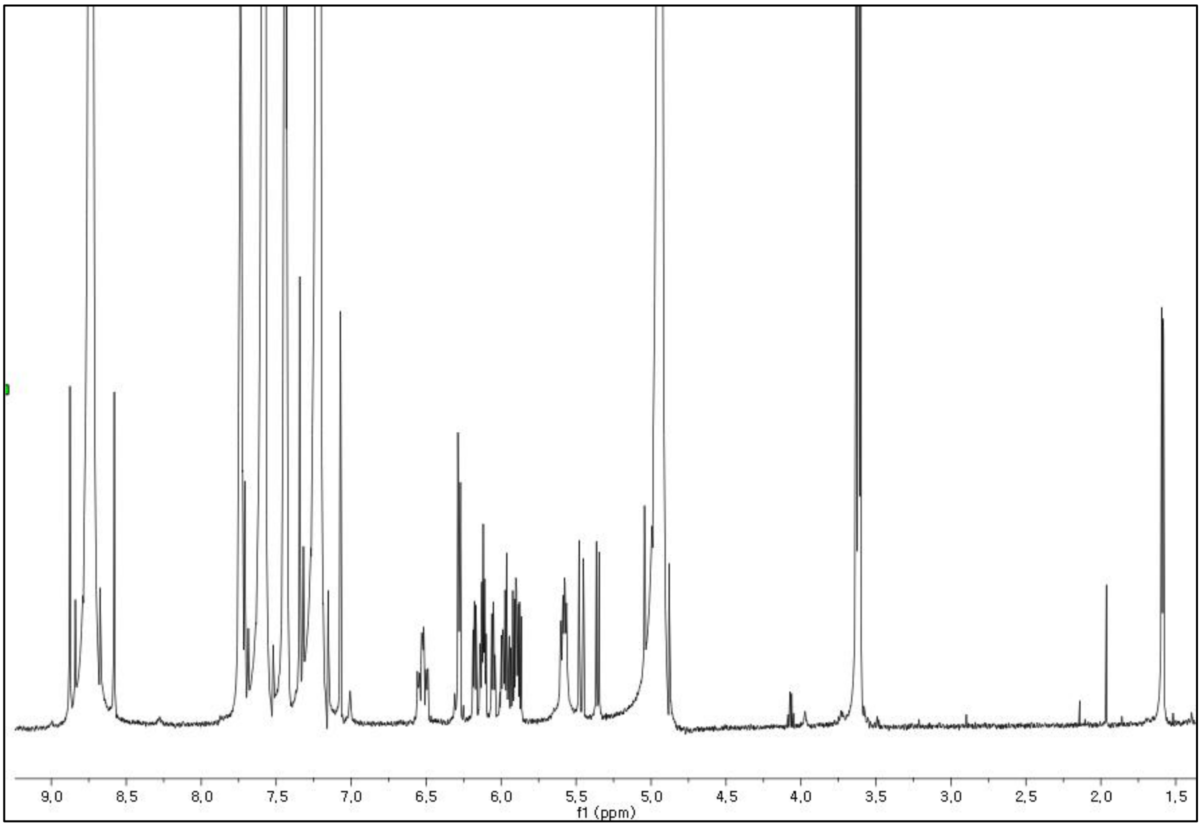

Figure S21. Phylogenetic tree based on 16S rDNA sequences of SNJ210 (1404bp).

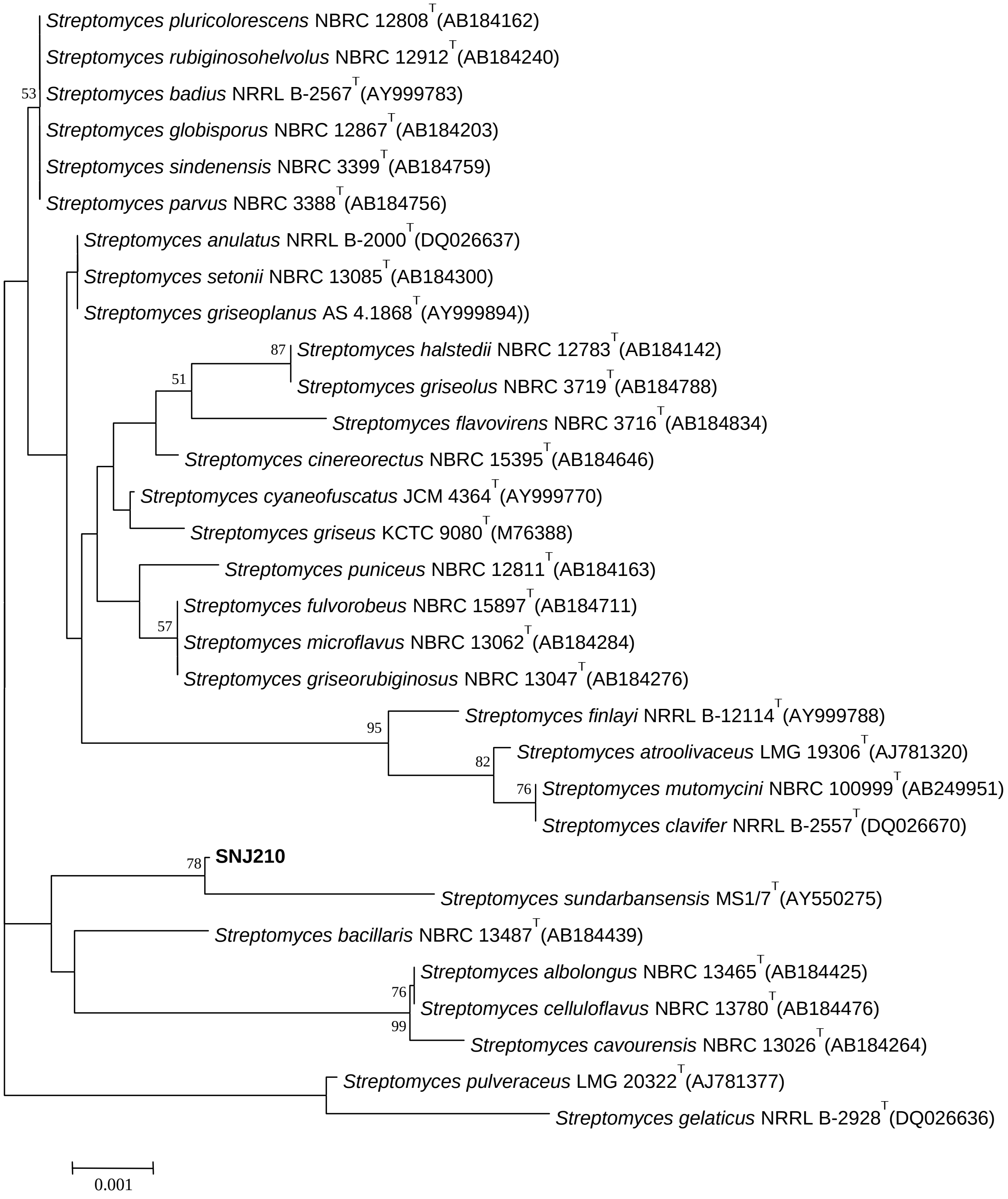

Figure S22. 16S rDNA sequence data of *Streptomyces* sp. SNJ210.

ATGCAAGTCGAACGATGAAATCACTTCGGTGGTGGATTAGTGGCGAACGGGTGAGTAAC
ACGTGGGCAATCTGCCCTTCACTCTGGGACAAGCCCTGGAAACGGGGTCTAATACCGGAT
ACCACTCTGTCCCGCATGGGACGGGGTTGAAAGCTCCGGCGGTGAAGGATGAGCCCGCG
GCCTATCAGCTTGTTGGTGGGGTAATGGCCTACCAAGGCGACGACGGGTAGCCGGCCTGA
GAGGGCGACCGGCCACACTGGGACTGAGACACGGCCCAGACTCCTACGGGAGGCAGCAG
TGGGGAATATTGCACAATGGGCGAAAGCCTGATGCAGCGACGCCGCGTGAGGGATGACG
GCCTTCGGGTTGTAAACCTCTTTCAGCAGGGAAGAAGCGCAAGTGACGGTACCTGCAGAA
GAAGCGCCGGCTAACTACGTGCCAGCAGCCGCGGTAATACGTAGGGCGCAAGCGTTGTCC
GGAATTATTGGGCGTAAAGAGCTCGTAGGCGGCTTGTCACGTCGGATGTGAAAGCCCGGG
GCTTAACCCCGGGTCTGCATTCGATACGGGCTAGCTAGAGTGTGGTAGGGGAGATCGGAA
TTCCTGGTGTAGCGGTGAAATGCGCAGATATCAGGAGGAACACCGGTGGCGAAGGCGGA
TCTCTGGGCCATTACTGACGCTGAGGAGCGAAAGCGTGGGGAGCGAACAGGATTAGATA
CCCTGGTAGTCCACGCCGTAAACGTTGGGAACTAGGTGTTGGCGACATTCCACGTCGTCG
GTGCCGCAGCTAACGCATTAAGTTCCCCGCCTGGGGAGTACGGCCGCAAGGCTAAAACTC
AAAGGAATTGACGGGGGCCCGCACAAGCAGCGGAGCATGTGGCTTAATTCGACGCAACG
CGAAGAACCTTACCAAGGCTTGACATATACCGGAAAGCATCAGAGATGGTGCCCCCCTTG
TGGTCGGTATACAGGTGGTGCATGGCTGTCGTCAGCTCGTGTCGTGAGATGTTGGGTTAA
GTCCCGCAACGAGCGCAACCCTTGTTCTGTGTTGCCAGCATGCCCTTCGGGGTGATGGGG
ACTCACAGGAGACTGCCGGGGTCAACTCGGAGGAAGGTGGGGACGACGTCAAGTCATCA
TGCCCCTTATGTCTTGGGCTGCACACGTGCTACAATGGCCGGTACAATGAGCTGCGATGC
CGCGAGGCGGAGCGAATCTCAAAAAGCCGGTCTCAGTTCGGATTGGGGTCTGCAACTCGA
CCCCATGAAGTCGGAGTTGCTAGTAATCGCAGATCAGCATTGCTGCGGTGAATACGTTCC
CGGGCCTTGTACACACCGCCCGTCACGTCACGAAAGTCGGTAACACCCGAAGCCGGTGGC
CCAACCCCTTGTGGGAGGGAGCTGTCGAAGGT
